# LncAABR07025387.1 Enhances Myocardial Ischemia/Reperfusion Injury *Via* miR-205/ACSL4-Mediated Ferroptosis

**DOI:** 10.3389/fcell.2022.672391

**Published:** 2022-02-02

**Authors:** Weixin Sun, Xiang Wu, Peng Yu, Qian Zhang, Le Shen, Jiandong Chen, Huaqin Tong, Manlu Fan, Haibo Shi, Xiaohu Chen

**Affiliations:** ^1^ Department of Cardiology, Yancheng TCM Hospital Affiliated to Nanjing University of Chinese Medicine, Yancheng, China; ^2^ Department of Cardiology, Jiangsu Province Hospital of Chinese Medicine, Nanjing, China; ^3^ Department of Cardiology, Affiliated Hospital of Nanjing University of Chinese Medicine, Nanjing, China; ^4^ First Clinical Medical College, Nanjing University of Chinese Medicine, Nanjing, China; ^5^ Department of Gerontology, Jiangsu Province Hospital of Chinese Medicine, Nanjing, China; ^6^ Department of Gerontology, Affiliated Hospital of Nanjing University of Chinese Medicine, Nanjing, China; ^7^ Department of Cardiology, Liyang City Hospital of TCM, Changzhou, China

**Keywords:** myocardial I/R injury, ferroptosis, miR-205, ACSL4, lncAABR07025387.1

## Abstract

Ferroptosis is associated with the pathology of myocardial ischemia/reperfusion (MI/R) injury following myocardial infarction, which is a leading cause of death worldwide. Although long noncoding RNAs (lncRNAs) are known to regulate gene expression, their roles in MI/R-induced ferroptosis remain unclear. In this study, we explored the lncRNA expression profiles in a rat model of MI/R injury and found that the novel lncRNA, lncAABR07025387.1, was highly expressed in MI/R-injured myocardial tissues and hypoxia/reoxygenation (H/R)-challenged myocardial cells. Silencing lncAABR07025387.1 improved MI/R injury *in vivo* and inhibited myocardial cell ferroptosis under H/R conditions. Bioinformatics analyses and luciferase, pull-down, and RNA-binding immunoprecipitation assays further revealed that lncAABR07025387.1 interacted with miR-205, which directly targeted ACSL4, a known contributor to ferroptosis. Furthermore, downregulating miR-205 reversed the ACSL4 inhibition induced by silencing lncAABR07025387.1. These findings suggest that, mechanistically, lncAABR07025387.1 negatively regulates miR-205 expression and subsequently upregulates ACSL4-mediated ferroptosis. In conclusion, this study demonstrates that lncAABR07025387.1 acts as a competing endogenous RNA during MI/R injury and highlights the therapeutic potential of lncRNAs for treating myocardial injury.

## Introduction

Myocardial ischemia/reperfusion (MI/R) injury is a common challenge when conducting reperfusion treatment for myocardial infarction as it can lead to several adverse cardiovascular outcomes, such as systolic myocardial dysfunction, cardiac electrophysiology disorder, and myocardial death (Eltzschig and Eckle, 2011; Ibanez et al., 2015; Han et al., 2017). Consequently, MI/R injury is a leading cause of death worldwide (Hausenloy and Yellon, 2016). Numerous studies have associated MI/R injury with complex molecular and pathophysiological processes, including cell apoptosis, autophagy, and inflammatory responses (Ma et al., 2011; Liao et al., 2012; Petz et al., 2019). However, current strategies for treating MI/R injury are limited, and it is essential to elucidate the underlying mechanisms and investigate novel reagents to protect myocardial cells against MI/R-induced injury.

Ferroptosis is an iron- and oxidation-dependent type of death that is characterized by increased levels of reactive oxygen species (ROS) and lipid peroxidation (Dixon et al., 2015). The iron-containing enzyme lipoxygenase produces lipid hydroperoxides to trigger ferroptosis, whose function relies on the activation of ACSL4-dependent lipid biosynthesis. Conversely, GPX4 usually serves as a central repressor of ferroptosis, which is regulated by the cystine–glutamate antiporter SLC7A11 (Dixon et al., 2015). Recent studies have revealed that ferroptosis is associated with MI/R injury. For example, some groups have reported an increase in lipid peroxidation and iron overload, which are crucial hallmarks of ferroptosis, during transient MI/R exposure (Conrad and Proneth, 2019; Fang et al., 2019). In addition, Xia et al. noted that excessive ROS accumulation in a diabetes MI/R model led to ferroptosis within the tissues. During ferroptosis, glutathione peroxidase 4 (GPX4) is inactivated and acyl-CoA synthetase long-chain fatty acyl-CoA synthetase 4 (ACSL4) is activated to accelerate myocardial cell death (Li et al., 2020). Ge et al. reported that ischemia could induce iron enrichment in the myocardium, thereby worsening the damage caused by MI/R (Song et al., 2021). Although the molecular mechanisms of MI/R-induced ferroptosis have been explored in detail, the role of long noncoding RNAs (lncRNAs) in this process remains unclear.

LncRNAs are a class of noncoding transcripts of more than 200 nucleotides that are distributed in the nucleus and cytoplasm with cell-specific expression and exert critical regulatory functions in multiple biological processes and diseases, including MI/R injury (Nagano and Fraser, 2011; Cabili et al., 2015; Huang et al., 2016; Liu et al., 2016). LncRNAs have been reported to affect target gene expression in many ways, including chromatin modification, transcriptional regulation, and posttranscriptional regulation (Chu et al., 2015; Kopp and Mendell, 2018). Intriguingly, accumulating evidence has indicated that lncRNAs can also function as competing endogenous RNAs (ceRNAs) that sponge microRNAs (miRNAs) to upregulate downstream genes (Thomson and Dinger, 2016). Although some studies have suggested that lncRNAs are involved in cell ferroptosis during the progression of cancers or the initiation of pulmonary fibrosis by interfering with the expression of target genes (Mao et al., 2018; Wang L. et al., 2019; Wu et al., 2020; Yang et al., 2020), the exact roles of lncRNAs in the heart under MI/R conditions have not yet been elucidated.

In this study, we explored the expression profile of lncRNAs in a rat model of MI/R injury and found that lncAABR07025387.1 was significantly upregulated in injured heart tissues. Moreover, lncAABR07025387.1 levels also increased in the hypoxia/reoxygenation (H/R)-exposed *in vitro* model. Subsequent functional studies demonstrated that lncAABR07025387.1 is involved in H/R-induced cell ferroptosis *in vitro* and tissue injury *in vivo*. Mechanistically, lncAABR07025387.1 served as a ceRNA to upregulate the expression of ACSL4, a ferroptosis contributor, by sponging miR-205. Thus, our findings reveal the function of the lncAABR07025387.1/miR-205/ACSL4 axis in the pathogenesis of MI/R injury and provide a novel therapeutic target for myocardial injury.

## Materials and Methods

### Animal Experiments

All animal experiments were approved by the Animal Care and Ethics Committee of the Jiangsu Province Hospital of Chinese Medicine and were designed according to ARRIVE guidelines (Supplementary Table S1). Adult male Sprague Dawley rats (3 months old, 200 g) purchased from the Animal Center of Nanjing University were housed at a controlled temperature of 18–22°C and humidity of 50–70% under a 12-h light/dark cycle with free access to food and water.

A total of 60 rats were prepared in this study (survival *n* = 6 per group), and the study lasted for 6 days (Supplementary Figure S2: schematic diagram of experimental animals grouped and surgically molded for survival). Before the experiments, humane endpoint criteria were established, including labored breathing, obvious loss of body weight, prostration, unresponsiveness to external stimuli, and a drop in body temperature (Kilkenny et al., 2010; Percie du Sert et al., 2020). When rats presented with one or more of these reactions, they were assumed to have reached the humane endpoint and were immediately euthanized. An animal specialist monitored the rats daily, and not a single rat reached the humane endpoints before the end of the experiment. Approximately 20% of the rats died of myocardial infarction during or after surgery and were immediately euthanized according to their vital signs.

Each rat was numbered blindly and randomly allocated to different groups using the Linux command “shuf.” The detailed model construction method is described in the supplementary material (Supplementary Figure S3: Flowchart of constructing the myocardial ischemia–reperfusion injury model in rats). Before the operation, the rats were fasted overnight with free access to water. At the beginning of the operation, the rats were intraperitoneally injected with chloral hydrate (0.5 ml/100 g) for sedation) and 5% additional first dose for unsatisfactory sedation and made to inhale isoflurane for anesthesia (4% for induction and 2% for maintenance, 0.5 L/min, a single-channel anesthesia machine, ABS-100, http://www.yuyanbio.com). After incising the skin, the muscle was carefully separated layer by layer, and the trachea was exposed and fixed locally with a self-made pull hook. The trachea was then cut with a 10 ml syringe needle. The anesthesia mask was then replaced with the tracheal intubation. The ventilator was connected to the anesthesia machine for isopentane inhalation (2%). Since this process of changing anesthesia takes about 2–3 min, the whole process of isoflurane anesthesia without chloral hydrate sedation may lead to intraoperative awareness and recovery, increasing the pain of experimental animals. The rats were exposed to left thoracotomy. The proximal left anterior descending artery (LAD) was ligated using a 6.0 Prolene suture to induce myocardial ischemia. A small semi-cylindrical plastic hose was inserted to the LAD to facilitate the opening of the knot during reperfusion and to reduce the mechanical damage to the heart tissue and blood vessels. About 5 min later, the chest cavity was temporarily closed with a non-damaging hemostatic clip. After 30 min of ischemia, the noninjury hemostatic clip was loosened, the ligation and the protective tube were removed for reperfusion, and the chest was closed. The rats in the sham group underwent the same operation without being subjected to MI/R (Akodad et al., 2017; Yang et al., 2020). Three hours after reperfusion, the rats were euthanized using cervical dislocation under isoflurane anesthesia. The serum was isolated, and the heart tissues were then removed for further study. Pathological and serological assays were used to evaluate the MI/R model. Myocardial enzyme leakages, such as LDH activity and CK-MB, were then measured using an LDH ELISA Kit (Abcam, MA, United States) and CK-MB ELISA Kit (Abcam, MA, United States), respectively, according to the manufacturer’s instructions. MI/R rats were subjected to different treatments by injection with vectors 2 days before the MI/R operation. The lncRNA AABR07025387.1 overexpression vector (lncAABR07025387.1-OE), empty vector (vector), siRNA (si-lncAABR07025387.1), and control siRNA (si-ctrl) were designed and obtained from GenePharma (Shanghai, China). For *in vivo* transfection, the molecules (50 μg) were mixed with *in vivo*-jetPEI reagent (Polyplus-transfection, SA, United States) according to the manufacturer’s protocol and injected into the left ventricle anterior wall of the rat myocardium 2 days before MI/R surgery (Fromes et al., 1999).

### Cell Culture and Treatment

The H9C2 and HEK293T cell lines were purchased from ATCC (Manassas, VA, United States) and maintained in Dulbecco’s modified Eagle’s medium (DMEM, Gibco, MD, United States) supplemented with 10% fetal bovine serum (FBS; Gibco) and 1% penicillin–streptomycin (Thermo, MA, United States). To generate the H/R model, the cells were maintained under hypoxic conditions (2% O_2_, 93% N_2_, and 5% CO_2_) for 2 h in a hypoxic incubator (YQX-II, Jianghong, Jiangsu, China) and then transferred to a normoxic incubator (5% CO_2_) for 4 h. Control cells were cultured in a normoxic incubator (Hu et al., 2016).

### Hematoxylin and Eosin Staining

For H&E staining (Fischer et al., 2008), myocardial tissues were fixed with 10% formaldehyde solution, embedded in paraffin, and cut into 5 µm sections that were stained using an H&E staining kit (Beyotime, China) according to the manufacturer’s instructions. Images were captured using a microscope (Nikon, Tokyo, Japan).

### 2,3,5-Triphenyltetrazolium Chloride Staining

Once the left and right atria and right ventricle had been removed from the heart, they were cut into 1.5 mm sections along an axis parallel to the atrioventricular groove. The sections were stained with 1% TTC solution at 37°C for 20 min, and the infarcted areas were analyzed using Image-Pro Plus 5.0 (Chang et al., 2016).

### Iron Assay

Myocardial tissues or cells were collected, and intracellular ferrous iron levels were detected using an iron assay kit (Abcam, MA, United States) (Wang M. et al., 2019). After being washed several times with PBS, the samples were homogenized, incubated with an iron reducer, and stained with an iron probe for 1 h. The iron content was determined at 593 nm using a microplate reader (Bio-Tek, WA, United States).

### ROS Detection

H9C2 cells were plated into six-well plates (10^6^ cells/well) and subjected to different treatments. The cells were then incubated with CellRox Green reagent (5 μM, Thermo) for 30 min, as described previously (Yokoyama et al., 2017). Nuclei were stained with 4,6-diamidino-2-phenylindole (DAPI), and the cells were imaged using a fluorescence microscope (Zeiss, Germany).

### qRT-PCR

Total RNA was isolated from cells or myocardial tissues using TRIzol reagent (Thermo) and reverse-transcribed into cDNA using a PrimeScript RT Master Mix Kit or PrimeScript miRNA cDNA Synthesis Kit (TaKaRa, Japan). The expression of genes, miRNAs, and lncRNAs was measured using a qRT-PCR system (Bio-Rad, CA, United States) using SYBR Green reagent (Bio-Rad). Data were obtained using the 2^−ΔΔCT^ method and normalized to U6 or GAPDH (Livak and Schmittgen, 2001). The primers used in this study are presented in Supplementary Table S2.

### Western Blot Analysis

Western blotting was performed as described previously (Mahmood and Yang, 2012). Briefly, cells or myocardial tissues were collected and homogenized, and total proteins were isolated using radioimmunoprecipitation assay buffer (Beyotime, China) according to the manufacturer’s protocol. Equal amounts of protein (20 µg/lane) from each sample were separated using SDS-PAGE and transferred onto polyvinylidene fluoride membranes (Bio-Rad). The membranes were blocked with 5% BSA solution for 2 h at 28°C, incubated with primary antibodies at 4°C overnight, and then hybridized with secondary antibodies at 28°C for 2 h. The membranes were stained using an ECL kit (Thermo) and analyzed using ImageJ software. GAPDH was used as an internal control. The following antibodies were used: primary antibodies against FTH1 (1:1,000, Abcam), GPX4 (1:1,000, Abcam), ACSL4 (1:1,000, Abcam), LPCAT3 (1:1,000, Abcam), PTGS2 (1:1,000, Abcam), NRF2 (phospho S40) (1:1,000, Abcam), HIF (1:1,000, Abcam), lamin B1 (1:5,000, Abcam), and GAPDH (1:5,000, Abcam) and secondary antibodies for anti-Rat IgG (HRP; 1:5,000, Abcam) and anti-mouse IgG (HRP; 1:5,000, Abcam).

### RNA-Seq

The RNA-Seq assay was performed in Decodegenomics Ltd., (http://www.dggene.com/). Myocardial tissues from sham or MI/R-injured rats (*n* = 3 per group) were washed with PBS. Total RNA was isolated using TRIzol reagent (Invitrogen), reverse-transcribed into cDNA to create a sequencing library using a Stranded RNA-Seq Library Prep Kit (Illumina), and analyzed using an Illumina HiSeq 4000 Sequencing System (Tsingke Biotechnology, China). Annotated lncRNAs were analyzed further, and differentially expressed lncRNAs were displayed as a heatmap using R package “pheatmap.”

### Northern Blot

Northern blotting was performed as described previously (Wang et al., 2013). Total RNA derived from H9C2 cells was separated using formaldehyde gel, transferred onto nylon membranes (Millipore, MA, United States), and hybridized with DIG-labeled probes for lncAABR07025387.1 at 60°C overnight. The lncAABR07025387.1 probe was synthesized using a DIG labeling kit (Roche, CA, United States). The membranes were then analyzed using a DIG luminescent detection kit (Sigma, MA, United States) according to the manufacturer’s protocol. A DIG-labeled U6 probe was used as an internal control.

### Fluorescence in *In Situ* Hybridization

Cy3-labeled lncAABR07025387.1 or 18s probes were obtained from Ribobio (Guangzhou, China). After prehybridization with 0.5% Triton X-100 for 10 min, H9C2 cells were incubated with the probes (5 μg/ml) overnight at 55°C, and their nuclei were stained with DAPI (Beyotime). Labeled probes were detected using a fluorescence microscope (Olympus, Tokyo, Japan) (Ranasinghe et al., 2018).

### Transfection

The miR-205 mimic, inhibitor, related controls, ACSL4 overexpressed vector (ACSL4-OE), and empty vector (pcDNA3.1) were purchased from GeneChem (Shanghai, China). H9C2 cells were plated into six-well plates (10^6^ cells/well) and transfected with these molecules (5 nM each) using Lipofectamine 3000 (Invitrogen) for 48 h according to the manufacturer’s instructions.

### Cell Counting Kit-8 Assay

After different treatments, the cells were seeded into 96-well plates (5,000 cells/well) with CCK-8 solution (Sigma) and incubated for 4 h. The absorbance of each sample was measured at 450 nm using a microplate reader (Bio-Tek) according to the manufacturer’s instructions.

### Pull-Down Assay

Biotinylated DNA probes were hybridized with lncAABR07025387.1 or related antisense RNA (NC) using the Biotin RNA Labeling Mix (Roche) and T7/SP6 RNA polymerase (Roche). After being purified using an RNeasy Mini Kit (Qiagen, Valencia, CA, United States), the probes were dissolved and incubated with streptavidin-coated magnetic beads (Sigma) at 25°C for 2 h. The cells were lysed using a buffer (Roche) for 10 min and incubated with the probe-coated beads at 4°C for 3 h. Bound RNAs were eluted and isolated for qRT-PCR analysis (Liu et al., 2018).

### RNA-Binding Immunoprecipitation

RIP assays were performed using an EZ-Magna RIP Kit (Sigma), according to the manufacturer’s protocol. H9C2 cell lysates were reacted in RIP buffer containing rabbit anti-Ago2 or anti-IgG antibody–hybridized beads (Abcam). ACSL4 and miR-205 expressions were detected using qRT-PCR.

### Luciferase Assay

ACSL4 and lncAABR07025387.1 3′-UTRs were amplified in pmiR-RB-REPORT vectors (Ribobio). The binding sites were mutated using a site-directed mutagenesis kit (NEB E0554, United States). HEK293T cells were plated into six-well plates at a density of 10^6^ cells per well and co-transfected with a reporter containing the wild-type or mutant plasmid and miR-205 mimic, inhibitor, or related control using Lipofectamine 3000 reagent (Ribobio). After 48 h, luciferase activity was detected using a Dual-Luciferase Assay System (Promega Corp., Madison, WI, United States) according to the manufacturer’s guidelines.

### Measurement of Echocardiography

Cardiac function and structure were assessed *via* MyLab™ Eight Platform (Esaote, Italy). Briefly, rats were anesthetized with isoflurane (5%) using ventilation equipment, with fur carefully removed on the left chest, and then two-dimensional echocardiographic measurements were obtained. The left ventricular internal diastolic and systolic diameter (LVIDd and LVIDs), together with the left ventricular ejection fraction and fractional shortening (LVEF and LVFS), was measured from M-mode tracing.

### Statistical Analysis

Data were analyzed using SPSS 16.0 (SPSS Inc., Chicago, IL) and presented as mean ± standard deviation (SD). Two-tailed Student’s *t*-tests were used to compare two groups, while one-way analysis of variance (ANOVA) with Tukey’s multiple comparison test was used to compare multiple groups. *p* values <0.05 were considered statistically significant.

## Results

### Ferroptosis Is Involved in MI/R Formation

**FIGURE 1:**
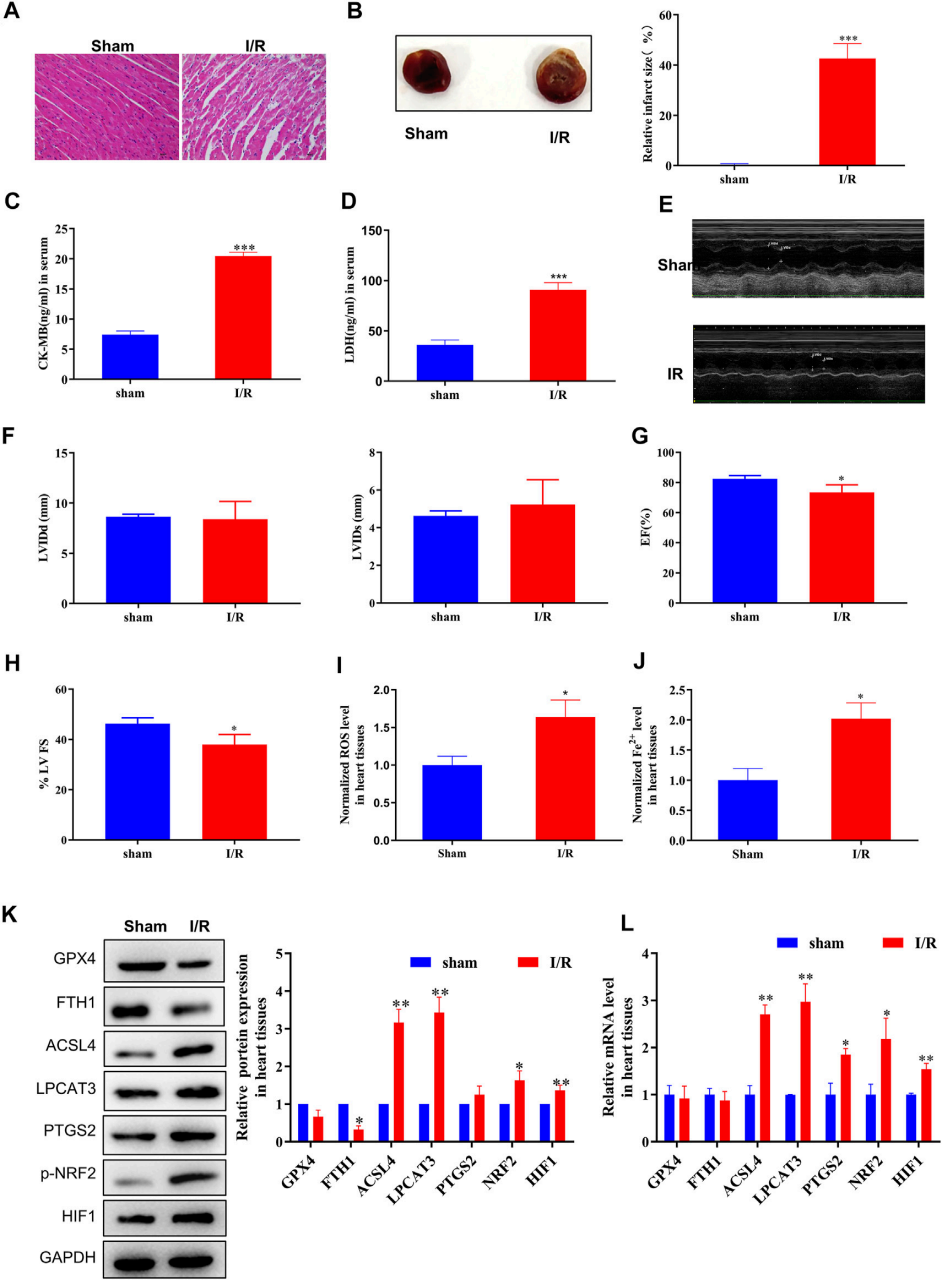
ACSL4–LPCAT3–mediated ferroptosis was induced in the MI/R model. **(A)** Representative H&E-stained images of the myocardium of sham or MI/R-injured rats. Bar = 200 µm. **(B)** TTC-stained infarct area. **(C,D)** Expression of myocardial enzyme leakage indicators, such as LDH and CK-MB, measured using ELISAs. **(E–H)** Measurement of echocardiography. **(I)** Level of ROS in myocardial tissues. **(J)** Concentration of iron in tissues measured using iron assays. **(K)** Representative blots and analyses of ferroptosis-related markers, including GPX4, FTH1, ACSL4, LPCAT3, PTGS2, NRF2 (phosphorylated), and HIF, in myocardial tissues from sham or injured rats. **(L)** Real-time PCR analysis of gene expression in rats subjected to MI/R surgery. Data represent mean ± SD (*n* = 3 per group). ^*^ *p* < 0.05, ^**^ *p* < 0.01, ^***^ *p* < 0.001 vs. the sham group.

First, we evaluated the rat model of MI/R injury using H&E staining, TTC staining, serological assays, and echocardiography. Following MI/R injury, myocardial cells underwent swelling, degeneration, and cardiac necrosis, with less transverse striations after H&E staining (Figure 1A). Hearts subjected to MI/R injury displayed a significantly larger infarct than the sham group (Figure 1B) and significant increases in serological biomarkers such as CK-MB and LDH (Figures 1C,D), verifying that the MI/R model was established successfully. In addition, the measurement of echocardiography showed a reduction in left ventricular ejection fraction, revealing that that MI/R model impaired cardiac function (Figures 1E–H).

After MI/R injury, we observed clear increases in both ROS and ferrous iron (Fe^2+^) levels in myocardial tissue compared to the sham group (Figures 1I,J). Moreover, increased levels of the cellular hypoxia inducible factor-1α (HIF-1α) and oxidative stress sensors NRF2 (phosphorylated) and endoperoxide-synthase-2 (PTGS2) confirmed the presence of hypoxia-induced oxidative conditions. Next, we measured the expression of ferroptosis biomarkers, finding that GPX4 and FTH1 protein levels were decreased and that the expression of ACSL4, NRF2 (phosphorylated), and LPCAT3 was dramatically upregulated in the MI/R group compared to the sham group (Figure 1K). Consistently, MI/R surgery significantly increased the mRNA levels of ACSL4, LPCAT3, PTGS2, and NRF2 (phosphorylated) (Figure 1L; Supplementary Figure S5). Taken together, these findings indicate that ferroptosis is induced during MI/R formation *in vivo*.

### LncRNA AABR07025387.1 Is Upregulated in MI/R-Injured Rats

**FIGURE 2:**
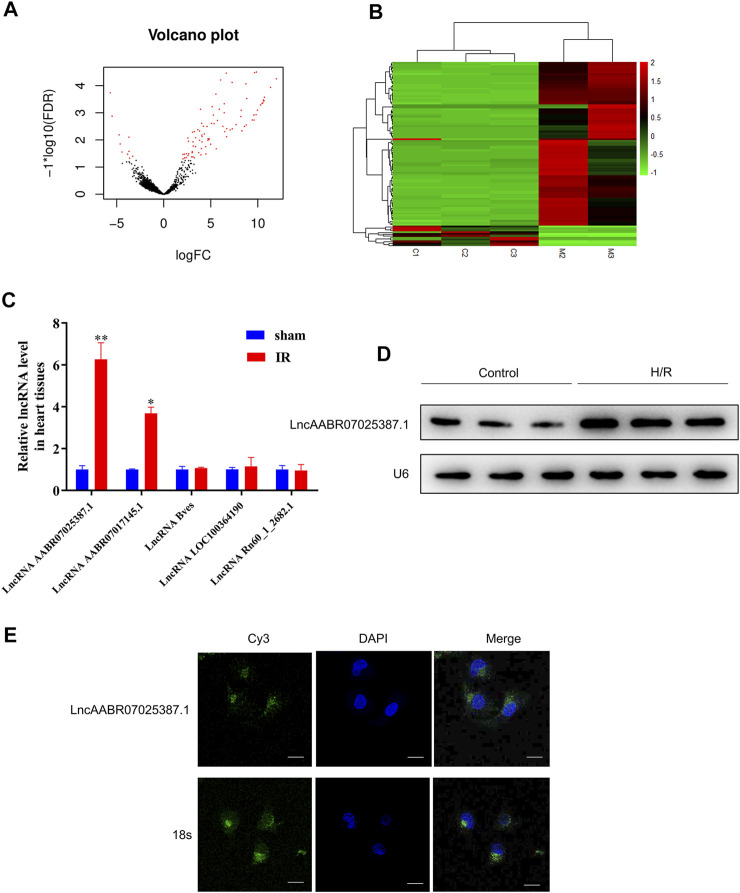
LncAABR07025387.1 was upregulated in the MI/R model. **(A)** Volcano graph showing differentially expressed lncRNAs (log fold change >2; *p* < 0.01). **(B)** Hierarchical clustering analysis of lncRNAs that were differentially expressed between normal (sham group) and MI/R-injured myocardial tissues (log fold change >2; *p* < 0.05; filtered to show the top 99 up- or downregulated lncRNAs). Values in red indicate upregulated lncRNAs, while those in green were downregulated. **(C)** Real-time PCR analysis of the top five upregulated lncRNAs in myocardial tissues from the MI/R group. Data represent mean ± SD (*n* = 3 per group). *p < 0.05, **p < 0.01 vs. the sham group. **(D)** Northern blot analysis of lncAABR07025387.1 in H9C2 cells exposed to H/R or normoxic (control) conditions. **(E)** RNA-FISH assays to detect the subcellular localization of lncAABR07025387.1 in H9C2 cells. Bar = 50 µm.

Next, we compared the lncRNA profiles of sham rats and MI/R-injured rats using high-throughput sequencing. As shown in Figures 2A,B, there were 99 differentially expressed lncRNAs, among which 88 were upregulated and 11 were downregulated in the myocardium of rats following MI/R treatment (Supplementary Table S3). The top five upregulated lncRNAs were lncAABR07025387.1, lncAABR07017145.1, lncBves, lncLOC100364190, and lncRn60_1_2682.1, which were selected and validated using qRT-PCR. Consistent with our sequencing data, lncAABR07025387.1 levels were elevated by around sixfold in MI/R-injured hearts compared to the sham group (Figure 2C).

To further investigate the mechanisms underlying the role of lncAABR07025387.1 in MI/R injury, we generated an *in vitro* model to stimulate MI/R by exposing myocardial cells (H9C2) to H/R conditions. Northern blotting confirmed that lncAABR07025387.1 was upregulated in H/R-exposed cells (Figure 2D), and FISH assays revealed that lncAABR07025387.1 was mainly expressed independently in the cytoplasm (green) rather than in the nucleus (blue; Figure 2E). Thus, these results suggest that lncAABR07025387.1 may be involved in the process of myocardial injury.

### LncAABR07025387.1 Inhibition Enhances Cell Viability and Suppresses ACSL4-Mediated Ferroptosis

**FIGURE 3:**
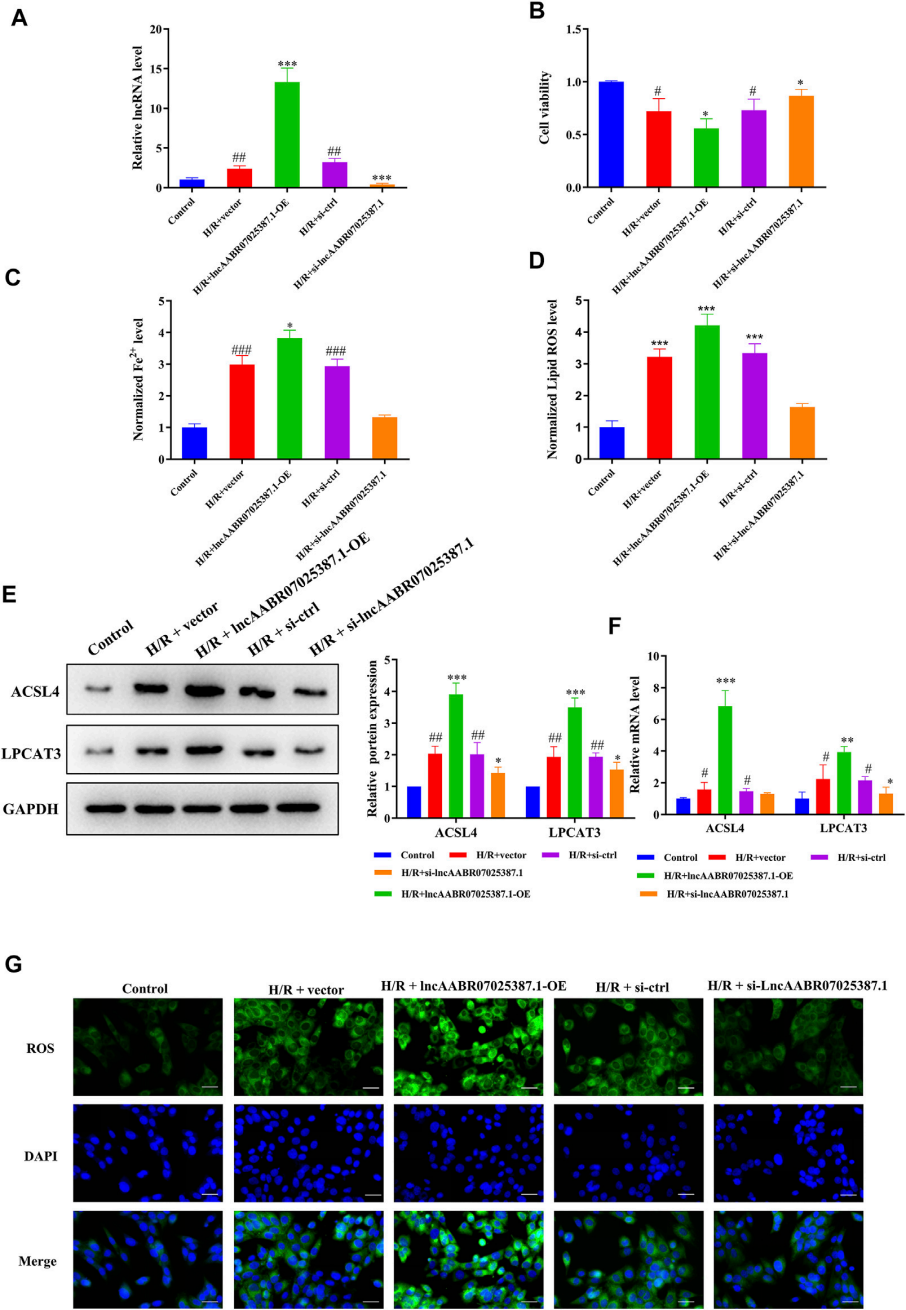
LncAABR07025387.1 correlated with ACSL4–LPCAT3–mediated ferroptosis *in vitro*. **(A)** LncAABR07025387.1-specific siRNA or overexpression vectors were transfected into H9C2 cells before H/R exposure. LncAABR07025387.1 expression was detected using qRT-PCR. **(B)** Cell viability was evaluated using CCK-8 assays 24 h after transfection. **(C)** Iron accumulation was monitored at 24 h in cells with lncAABR07025387.1 overexpression or downregulation, followed by H/R exposure. **(D)** Level of ROS in myocardial tissues after transfection. **(E)** Representative blots and analyses of ACSL4 and LPCAT3 in H/R-exposed cells after transfection. **(F)** Real-time PCR analysis of gene expression in cells with different treatments. **(G)** Intracellular ROS levels measured using fluorescence assays. Bar = 50 µm. Data represent the mean ± SD of three independent experiments. ^*^ *p* < 0.05, ^**^ *p* < 0.01, ^***^ *p* < 0.001 vs. the H/R + vector or H/R + si-ctrl groups. ^#^ *p* < 0.05, ^##^ *p* < 0.01, ^###^ *p* < 0.001 vs. the control group.

To confirm the role of lncAABR07025387.1 in MI/R injury, we designed specific siRNAs and overexpression plasmids and transfected them into myocardial cells before H/R treatment. As expected, transfection with the lncAABR07025387.1-OE plasmid clearly increased the expression of lncAABR07025387.1, whereas transfection with si-lncAABR07025387.1 markedly reduced its expression (Figure 3A). CCK-8 assays revealed that cells subjected to H/R conditions exhibited lower viability than the control group; however, lncAABR07025387.1 overexpression negated this effect, and siRNA-mediated silencing increased cell viability following H/R exposure (Figure 3B). Consistently, lncAABR07025387.1 upregulation dramatically increased lipid ROS and Fe^2+^ levels, whereas silencing lncAABR07025387.1 decreased the levels of these factors (Figures 3C,D). In addition, we found that lncAABR07025387.1 levels correlated positively with the protein and mRNA levels of ACSL4 and LPCAT3 (Figures 3E,F). Furthermore, higher ROS levels were observed in lncAABR07025387.1-overexpressing cells than in vector-transfected cells, while knocking down this lncRNA significantly inhibited ROS generation compared to the si-ctrl group (Figure 3G). These results indicate that lncAABR07025387.1 plays a key role in H/R-induced ferroptosis in myocardial cells.

### LncAABR07025387.1 Functions as a ceRNA

**FIGURE 4:**
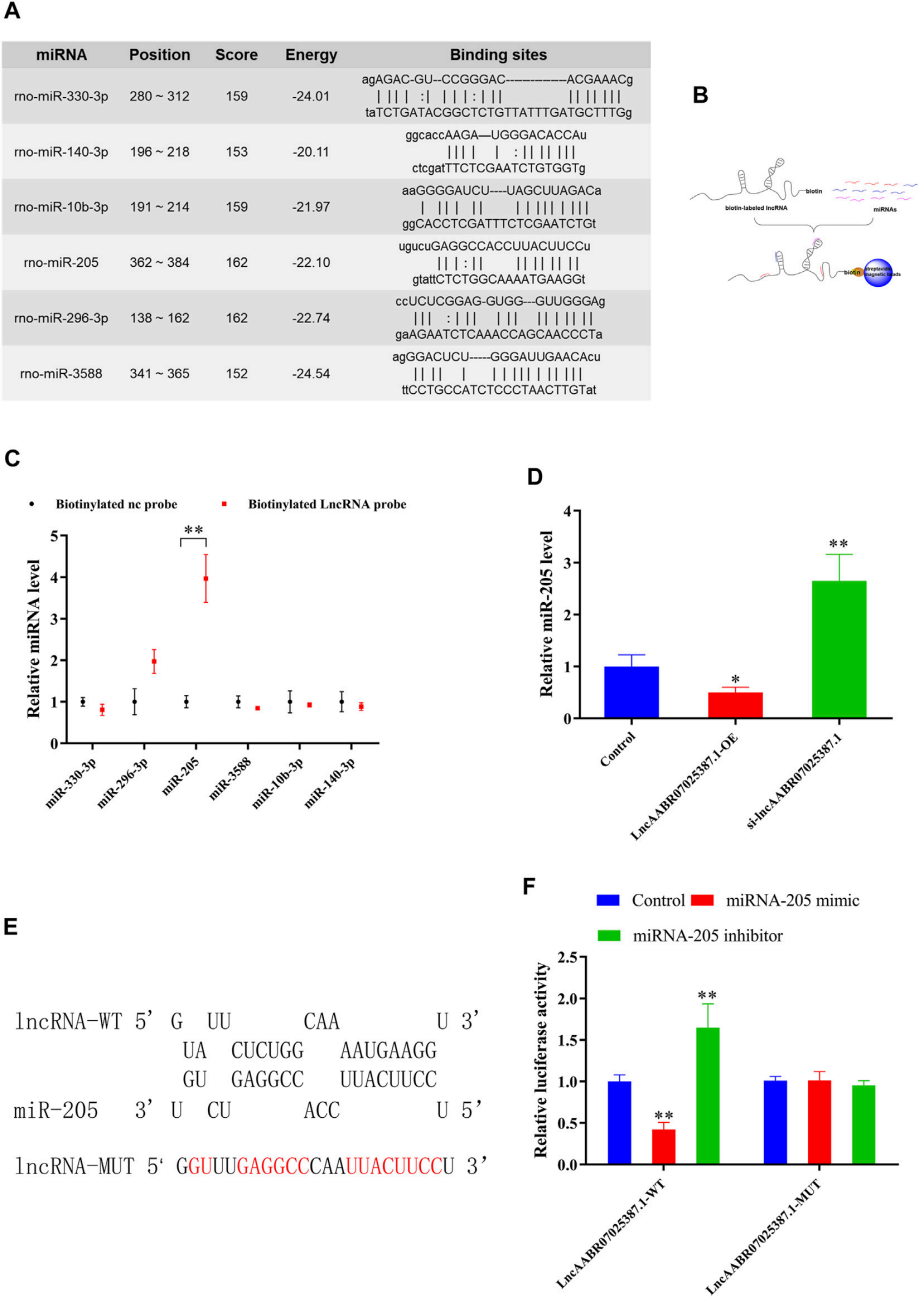
LncAABR07025387.1 interacts with and negatively regulates miR-205. **(A)** Potential binding sites of lncAABR07025387.1 with miR-330-3p, miR-296-3p, miR-205, miR-3588, miR-10b-3p, and miR-140-3p, as predicted by miRanda. **(B)** Schematic diagram of the RNA pull-down assay. **(C)** Expression of miRNAs targeted by lncAABR07025387.1 measured using qRT-PCR. **p < 0.01 vs. the biotinylated NC-probe group. **(D)** Real-time PCR analysis of lncAABR07025387.1 expression in H9C2 cells transfected with siRNA or overexpression vectors. **(E)** Predicted binding sites of miR-205 in the lncAABR07025387.1 transcript. **(F)** Luciferase activities were determined in HEK293T cells co-transfected with luciferase reporters containing a wild-type or mutant lncAABR07025387.1 3′-UTR and an miR-205 mimic or inhibitor. Luciferase activity was normalized to Renilla luciferase. Data represent the mean ± SD of three independent experiments. *p < 0.05, **p < 0.01 vs. the control group.

Next, we examined the molecular basis underlying the effect of lncAABR07025387.1 on cell ferroptosis, since interactions with miRNAs are a classical mechanism of lncRNA regulation. Using an online bioinformatics database (miRanda, http://www.miranda.org), we found that miR-330-3p, miR-296-3p, miR-205, miR-3588, miR-10b-3p, and miR-140-3p had a strong binding potential with lncAABR07025387.1 (Figure 4A). To verify these miRNAs, we performed biotin-labeled pull-down assays (Figure 4B) which revealed that lncAABR07025387.1-coated pellets were enriched with miR-205, but no other miRNAs, when compared to the control (Figure 4C).

To determine whether lncAABR07025387.1 could regulate miR-205 expression, we transfected H9C2 cells with si-lncAABR07025387.1 or lncAABR07025387.1-OE vector. qRT-PCR assays revealed that miR-205 expression was dramatically upregulated in cells with lncRNA knockdown and significantly downregulated in cells overexpressing this lncRNA (Figure 4D). We then confirmed the relationship between lncAABR07025387.1 and miR-205 using a luciferase assay with the binding sites predicted using the RNAhybrid database (http://www.bibiserv.cebitec.uni-bielefeld.de/rnahybrid) (Figure 4E). HEK293T cells transfected with wild-type lncRNA displayed a lower luciferase activity when co-transfected with an miR-205 mimic and a higher luciferase activity when treated with an miR-205 inhibitor. No obvious changes were observed in cells transfected with the mutated lncRNA 3′-UTR (Figure 4F). These results indicated that lncAABR07025387.1 could serve as a ceRNA, sponging miR-205 in cells and regulating molecular signaling pathways.

### miR-205 Directly Targets ACSL4

**FIGURE 5:**
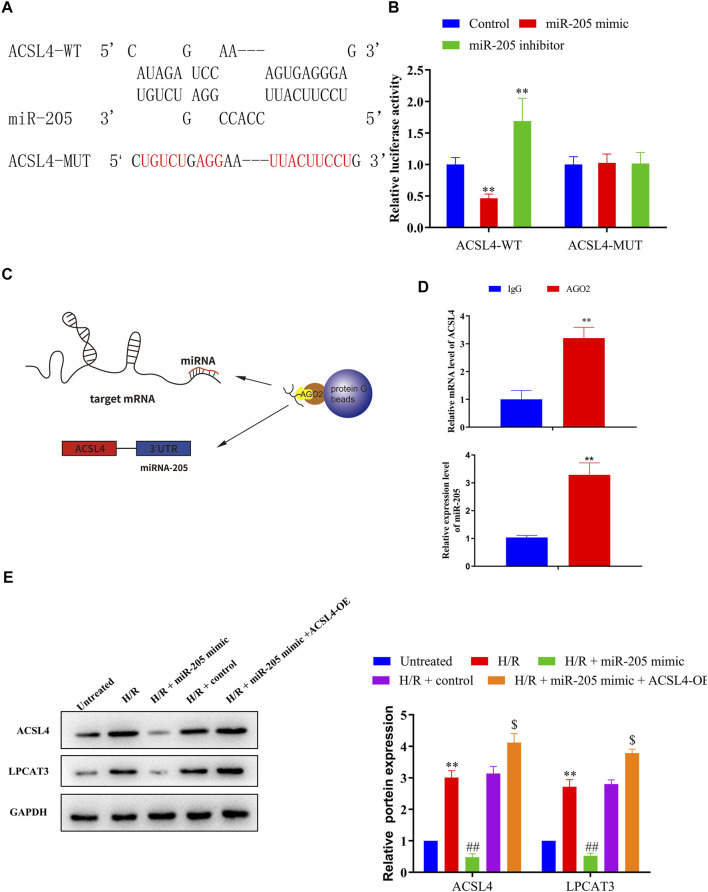
ACSL4 is a target gene of miR-205. **(A)** Predicted binding sites of miR-205 in the ACSL4 transcript. **(B)** Luciferase activities were determined in HEK293T cells co-transfected with luciferase reporters containing a wild-type or mutant ACSL4 3′-UTR and an miR-205 mimic or inhibitor. Luciferase activity was normalized to Renilla luciferase. **p < 0.01 vs. the control group. **(C)** Schematic diagram of the RIP assay. **(D)** Immunoprecipitated mRNA was isolated and the levels of ACSL4 and miR-205 were measured using qRT-PCR. **(E)** Representative blots and analyses of ACSL4 and LPCAT3 in H/R-exposed cells after miR-205 mimic transfection with or without the ACSL4 overexpression vector. ^**^ *p* < 0.01 vs. the untreated group. ^##^ *p* < 0.01 vs. the H/R + control group. ^$^ *p* < 0.05 vs. the H/R + miR-205 mimic group.

Bioinformatics analyses of miR-205 target genes (TargetScan, http://www.targetscan.org) revealed that ACSL4 was a possible downstream target gene of miR-205 (Figure 5A); therefore, we conducted luciferase assays to confirm putative binding sites. Co-transfection with the wild-type ACSL4 3′-UTR and miR-205 mimic reduced luciferase activity compared to the control group, whereas treatment with the miR-205 inhibitor increased luciferase activity. Notably, binding site mutation abolished the effects of the miR-205 mimic and inhibitor (Figure 5B). To confirm the interaction between miR-205 and ACSL4, we performed RIP assays (Figure 5C). Higher ACSL4 and miR-205 RNA levels were observed in Ago2 immunoprecipitates compared to control IgG immunoprecipitates (Figure 5D), suggesting that miR-205 could directly target ACSL4.

**FIGURE 6:**
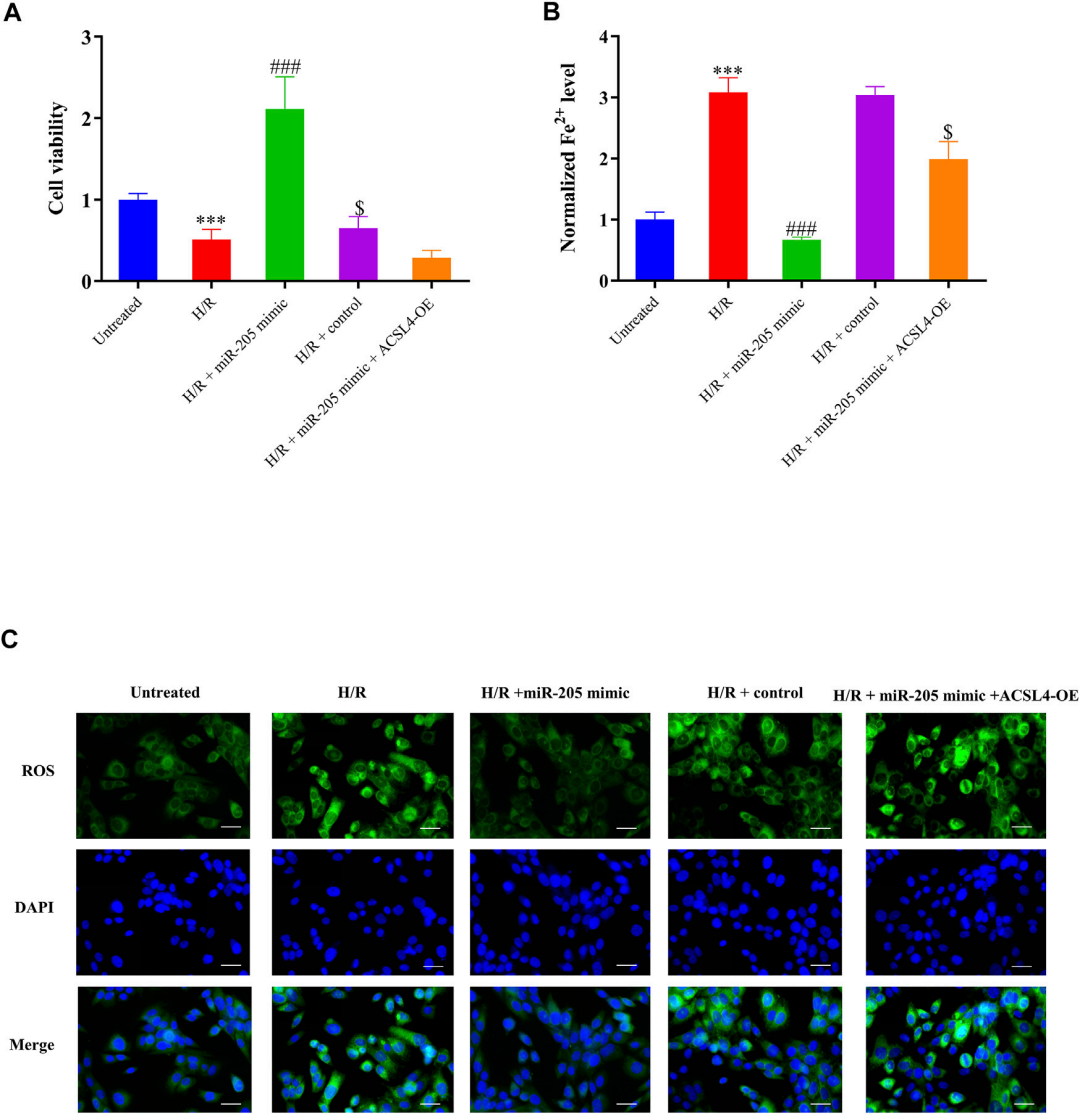
miR-205 overexpression inhibited cell ferroptosis and enhanced cell survival under H/R conditions by regulating ACSL4. **(A)** Cell proliferation was evaluated using CCK-8 assays 24 h after transfection. **(B)** Iron accumulation was monitored at 24 h in miR-205–overexpressing cells with or without ACSL4 overexpression followed by H/R exposure. **(C)** Intracellular ROS levels measured using fluorescence assays. Bar = 50 µm. Data represent the mean ± SD of three independent experiments. ^***^ *p* < 0.001 vs. the untreated group. ^###^ *p* < 0.001 vs. the H/R + control group. ^$^ *p* < 0.05 vs. the H/R + miR-205 mimic group.

Although miR-205 overexpression dramatically decreased ACSL4 and LPCAT3 expressions in cells under H/R exposure, their expression was recovered by ACSL4 overexpression (Figure 5E). CCK-8 assays further revealed that transfection with the ACSL4-OE vector eliminated the increase in cell viability caused by miR-205 overexpression compared to the H/R + control group (Figure 6A). Consistently, the accumulation of iron and ROS induced by H/R treatment was downregulated by miR-205 overexpression but was significantly reversed by co-transfection with the ACSL4 overexpression vector (Figures 6B,C). Taken together, we found that ACSL4 was the target of miR-205, involved in the regulation of the oxidative status of myocardial cells under H/R conditions.

### LncAABR07025387.1 Regulates Cell Viability and ACSL4-Mediated Ferroptosis via miR-205 *In Vitro*

**FIGURE 7:**
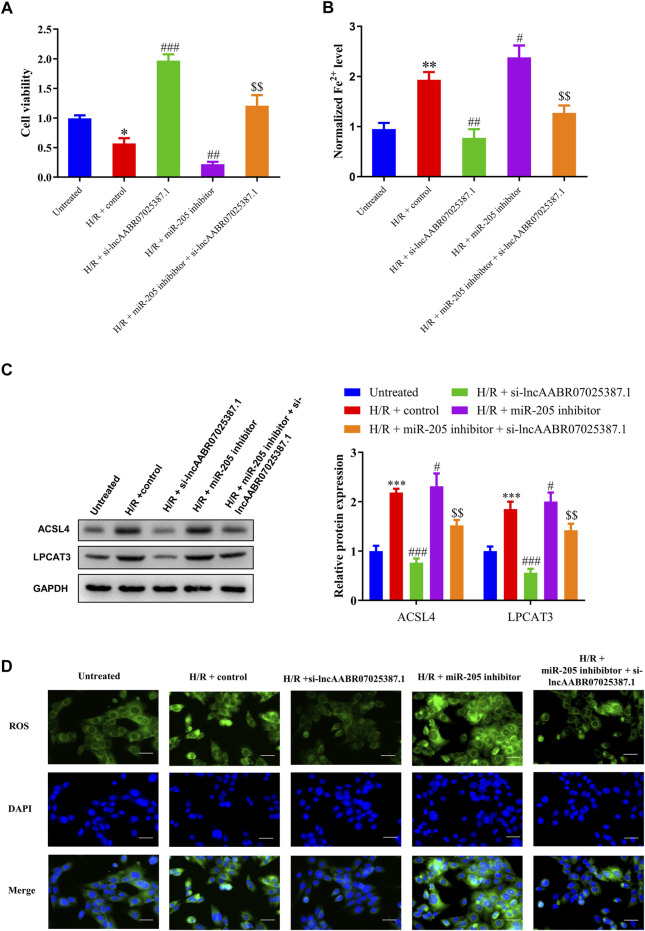
Silencing lncAABR07025387.1 inhibited H/R-induced ferroptosis in cells by regulating the miR-205/ACSL4 axis. **(A)** Cell proliferation was evaluated using CCK-8 assays 24 h after transfection. **(B)** Iron accumulation was monitored at 24 h in lncAABR07025387.1-inhibited cells with or without miR-205 downregulation followed by H/R exposure. **(C)** Representative blots and analyses of ACSL4 and LPCAT3 in H/R-exposed cells after silencing lncAABR07025387.1 with or without miR-205 inhibition. **(D)** Intracellular ROS levels measured using DCFH-DA fluorescence. Bar = 50 µm. Data represent the mean ± SD of three independent experiments. ^*^ *p* < 0.05, ^**^ *p* < 0.01, ^***^ *p* < 0.001 vs. the untreated group. ^#^ *p* < 0.05, ^##^ *p* < 0.01, ^###^ *p* < 0.001 vs. the H/R + control group. $$*p* < 0.01 vs. the H/R + miR-205 inhibitor group.

Next, we examined the role of the lncAABR07025387.1/miR-205/ACSL4 axis in the process of ferroptosis in myocardial cells under H/R conditions by treating them with lncAABR07025387.1 si-RNA or miR-205 inhibitors. After H/R exposure, lncAABR07025387.1 reversed the reduction in cell viability induced by H/R exposure compared to the control group (H/R + control) while also decreasing Fe^2+^ and ROS levels. Western blot assays showed that lncAABR07025387.1 knockdown markedly decreased ACSL4 and LPCAT3 levels compared to the H/R + control group; however, miR-205 inhibition partly enhanced the effect of H/R, inhibited cell viability, induced Fe^2+^ and ROS accumulation, and activated the expression of ferroptosis markers (Figures 7A–D). Moreover, miR-205 downregulation efficiently reversed the inhibition of ACSL4-mediated ferroptosis and increased cell viability induced by si-lncAABR07025387.1 in H/R-injured cells (Figures 7A–D). Taken together, these findings indicate that lncAABR07025387.1 promotes cell ferroptosis *via* the miR-205/ACSL4 axis.

### LncAABR07025387.1 Regulates the Process of MI/R Injury *In Vivo*

**FIGURE 8:**
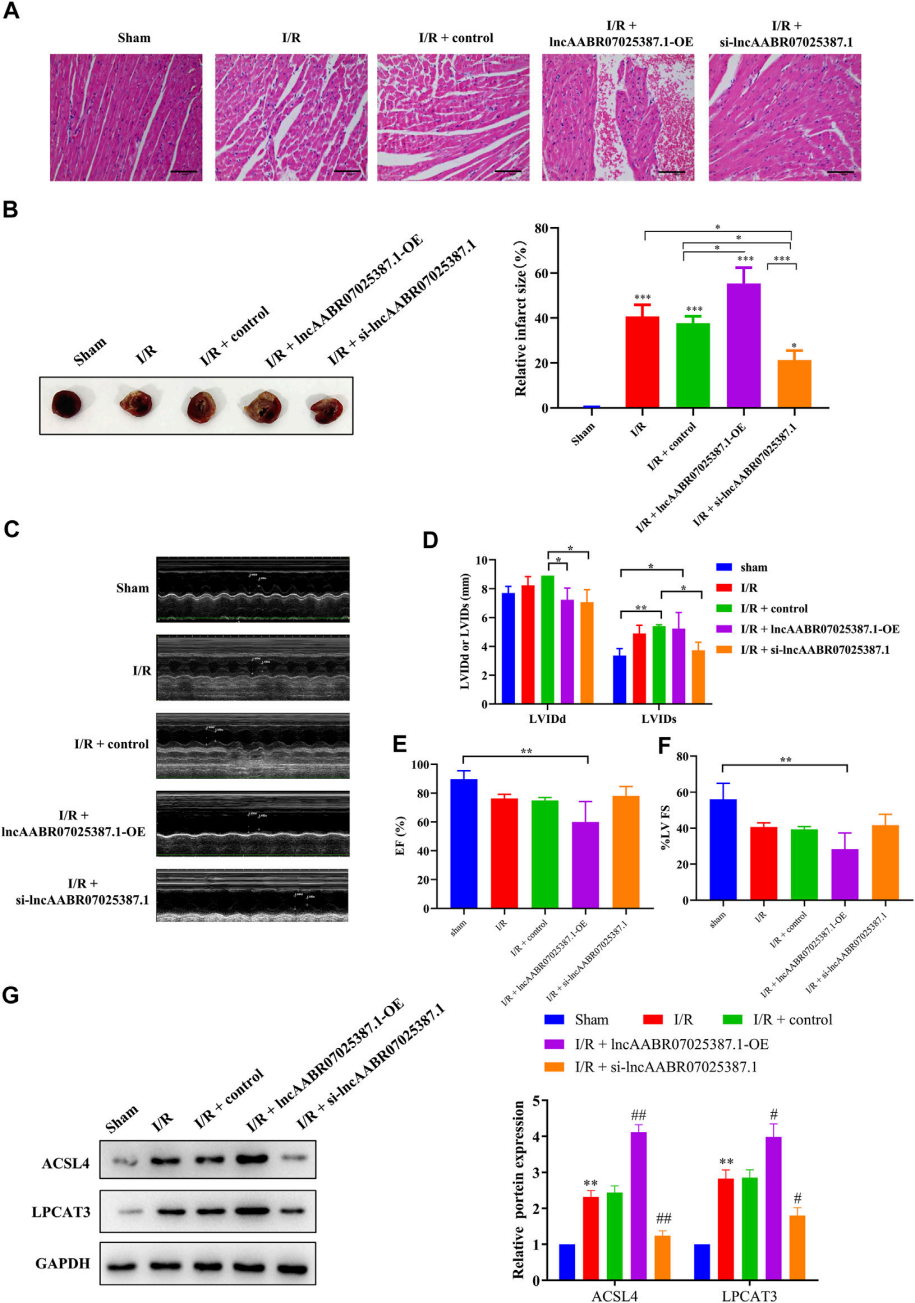
LncAABR07025387.1 knockdown alleviated MI/R injury *in vivo*. **(A)** Representative images of H&E staining in the myocardium of transfected sham or MI/R-injured rats. Bar = 200 µm. **(B)** TTC-stained infarct area. **(C–F)** Measurement of echocardiography in the myocardium of transfected sham or MI/R-injured rats. **(G)** Representative blots and analyses of ACSL4 and LPCAT3 in heart tissues after transfection with si-lncAABR07025387.1 or lncAABR07025387.1 overexpression vectors. Data represent mean ± SD (*n* = 3 per group). ^**^ *p* < 0.01 vs. the sham group. ^#^ *p* < 0.05, ^##^ *p* < 0.01 vs. the I/R + control group.

Finally, we confirmed the role of lncAABR07025387.1 in the progression of MI/R injury *in vivo*. LncAABR07025387.1 overexpression markedly enhanced the degree of injury in the myocardial tissues of MI/R rats, whereas silencing this lncRNA significantly alleviated MI/R injury (Figures 8A–F). Western blot assays further confirmed that silencing lncAABR07025387.1 decreased the expression levels of ACSL4 and LPCAT3 in the myocardium of rats subjected to MI/R surgery, whereas the overexpression of lncAABR07025387.1 enhanced the expression of these biomarkers (Figure 8G). The results showed that lncAABR07025387.1 regulated the process of MI/R injury *in vivo*.

## Discussion

Ferroptosis is an important form of myocardial cell death that occurs during the progression of MI/R injury (Li et al., 2020). In this study, we found that rats subjected to MI/R injury displayed increased ROS production and Fe^2+^ deposition that was reduced by injection with Fer-1 (Supplementary Figure S1: Fer-1 alleviated MI/R injury in rats). Consistently, MI/R-injured rats had decreased expression of ferroptosis inhibitors, such as GPX4 and FTH1, and increased expression of ferroptosis contributor markers, including ACSL4 and LPCAT3, confirming that ferroptosis occurred in our MI/R model, which agrees with the findings reported previously (Dixon et al., 2015; Muhoberac and Vidal, 2019; Macias-Rodriguez et al., 2020). Similar results were also obtained in H/R-exposed myocardial cells.

The I/R injury involves aberrant regulation of the mitochondrial function, apoptotic and autophagic pathways, and signal transducers. Long noncoding RNAs (lncRNAs, >200 nt) are typical noncoding RNAs, which have been proved previously to be closely associated with MI/R injury (Ghafouri-Fard et al., 2020). Many noncoding RNAs have been proved to play crucial roles in regulating MI/R injury, either alleviating the injury or aggravating it, constituting potential therapeutic targets (Gomes et al., 2017). For instance, lncRNA MALAT1 was involved in the cardiomyocyte apoptosis process (Cheng et al., 2020), while lncRNA AK139328 regulated autophagy during MI/R injury (Yu et al., 2018). In this study, we identified 99 dysregulated lncRNAs in the MI/R-injured myocardium and found that the high expression level of lncAABR07025387.1, a novel lncRNA that has not been reported before, was associated with ferroptosis in myocardial cells suffering from I/R injury. Gain- and loss-of-function experiments further revealed that lncAABR07025387.1 could promote H/R-induced myocardial cell ferroptosis, as evidenced by increased ROS production, intracellular Fe^2+^ accumulation, and the dysregulation of ferroptosis-associated markers. Together, these findings support the hypothesis that lncAABR07025387.1 is an important regulator of ferroptosis during the progression of MI/R injury. Previous studies have reported that the functional role of lncRNAs depends on their patterns of subcellular localization (Chen, 2016; Carlevaro-Fita and Johnson, 2019). Among all mechanisms behind the role of lncRNA located in the cytoplasm in MI/R injury, the miRNA/targeting axis ranked the top. Since RNA-FISH assays revealed that lncAABR07025387.1 was localized in the cytoplasm of H9C2 cells, this lncRNA might affect the expression of its target genes at the posttranscriptional level in the cytoplasm, sponging miRNAs to serve as a competing RNA to regulate the target genes (Paraskevopoulou and Hatzigeorgiou, 2016). For instance, by regulating the miR-29a/SIRT1/AMPK/PGC1 axis, lncRNA Oip5-as1 attenuates MI/R injury (Niu et al., 2020). LncRNA PEAMIR sponged miR-29b-3p to alleviate apoptosis and inflammatory response of PM2.5-aggravated MI/R injury (Pei et al., 2020). The overexpression of lncRNA Oprm1 benefited MI/R injury by competing with miR-30b-5p to increase endogenous H_2_S (Hu et al., 2020). Also, lncRNA TUG1 was associated with autophagy regulation during MI/R injury via targeting the miR-142-3p/HMGB1 and Rac1 axis (Su et al., 2019). In this work, RNA pull-down assays revealed that lncAABR07025387.1 could pull down miR-205 and that lncAABR07025387.1 expression correlated negatively with miR-205, confirming that this lncRNA sponges miR-205. Therefore, we revealed that lncAABR07025387.1 regulated ferroptosis during the progression of MI/R injury through miR-205.

miR-205 was a widely studied miRNA in cancers, with dual roles depending on the tumor microenvironment (Qin et al., 2013). For example, in prostate carcinoma, miR-205 repressed key oncogenic pathways (Boll et al., 2013). It also inhibited cell proliferation of pancreatic cancer stem cells (Chaudhary et al., 2017). Conversely in endometrial cancer, miR-205 is associated with poor patient overall survival (Karaayvaz et al., 2012). No studies have yet reported a relationship between miR-205 and MI/R injury. Here, we determined that miR-205 could alleviate I/R-induced myocardial ferroptosis by interfering with ACSL4 expression which agreed with the previous report. miR-205 inhibits the expression of ACSL4 by targeting its 3′-UTR, and anti-miR-205 can accelerate lipogenesis through regulating ACSL4 in hepatoma cells (Cui et al., 2014). As a lipid metabolism–related gene, ACSL4 is an essential mediator of ferroptosis, and its upregulation has been reported to increase the oxidation of fatty acids, thus contributing to ferroptosis (Xie et al., 2016). Chen et al. identified that suppressing ACSL4 levels could be beneficial for spinal cord injury repair by inhibiting ferroptosis (Zhou et al., 2020). Consistently, the bioinformatic analysis and luciferase assays conducted in this study revealed that miR-205 directly targets ACSL4 to improve MI/R-induced injury in myocardial cells. Moreover, we found that lncAABR07025387.1 could positively control ACSL4 expression by binding miR-205 in H9C2 cells, thereby promoting ferroptosis and aggravating myocardial injury. Since one miRNA can regulate several target genes, while individual genes may be controlled by many lncRNAs, further studies are required to understand the biological conditions that may affect ferroptosis in MI/R-challenged myocytes.

These are some limitations in our study. First, due to equipment limitation, we did not observe cell morphology using a transmission electron microscope, especially mitochondrial morphology in the MI/R heart tissues. Second, echocardiography was less sensitive to myocardial ischemia/reperfusion injury, although we found a trend in ejection fraction in our study. Cardiac magnetic resonance (CMR) could provide a comprehensive, multi-angle view of the heart, especially the method of late gadolinium enhancement (LGE). In future studies, we will try to use this technique called myocardial biopsy to further explore the mechanisms of myocardial ischemia/reperfusion injury.

In summary, we demonstrated that the novel lncRNA lncAABR07025387.1 plays a critical role in ferroptosis in MI/R-challenged myocardial cells by regulating the miR-205/ACSL4 axis. Together, our findings provide in-depth insights into the formation of MI/R injury and provide a potential therapeutic target for ferroptosis.

## Data Availability

The datasets presented in this study can be found in online repositories. The names of the repository/repositories and accession number(s) can be found at: https://www.ncbi.nlm.nih.gov/bioproject/PRJNA743436.
