# Supplementary figures and images for: LncAABR07025387.1 Enhances Myocardial Ischemia/Reperfusion Injury Via miR-205/ACSL4-Mediated Ferroptosis
Source: Front Cell Dev Biol. 2022 Feb 2;10:672391. doi: 10.3389/fcell.2022.672391 (PMC8847229; doi:10.3389/fcell.2022.672391)

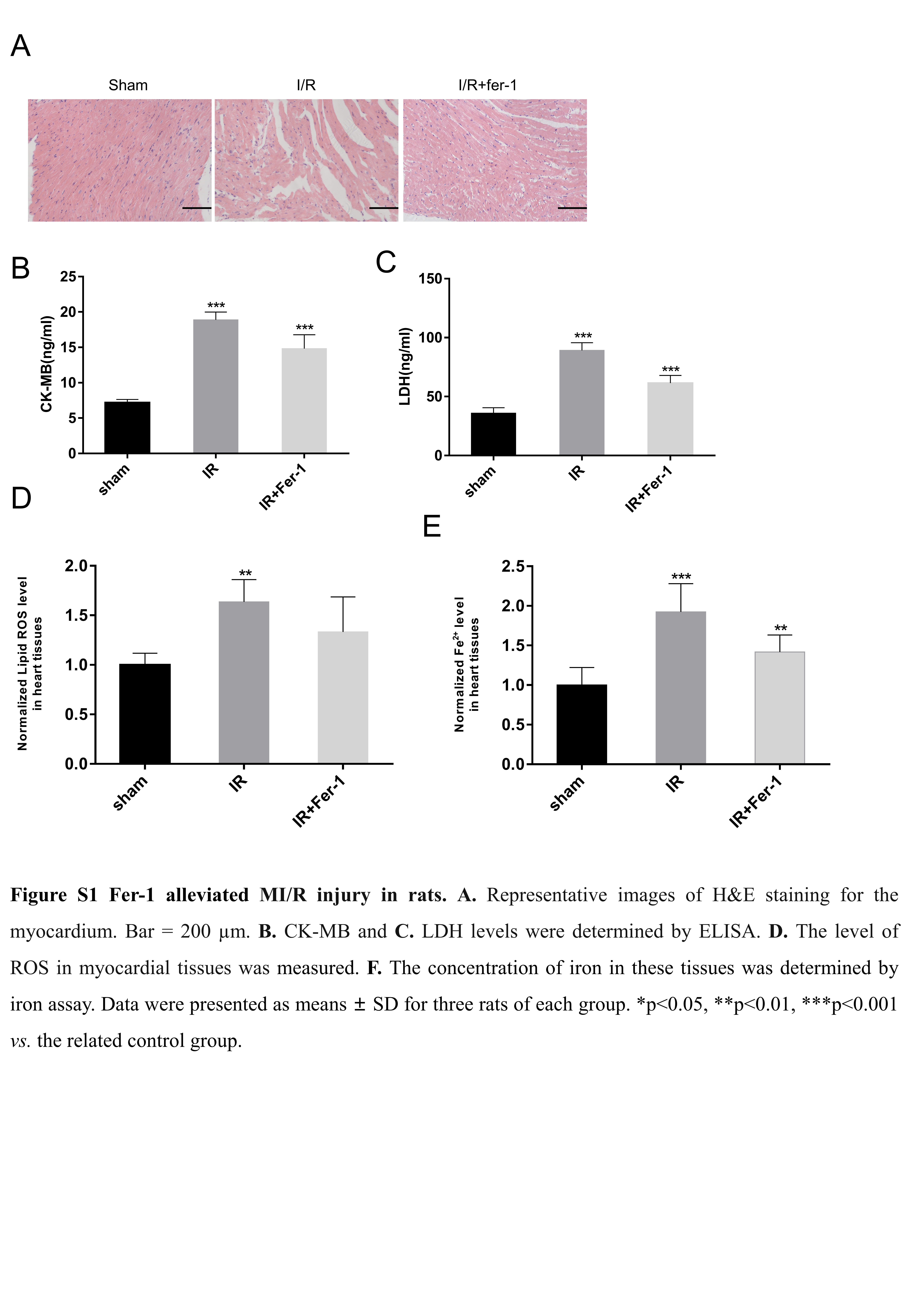

Supplement: Supplementary file 2 [file Image1.TIF]

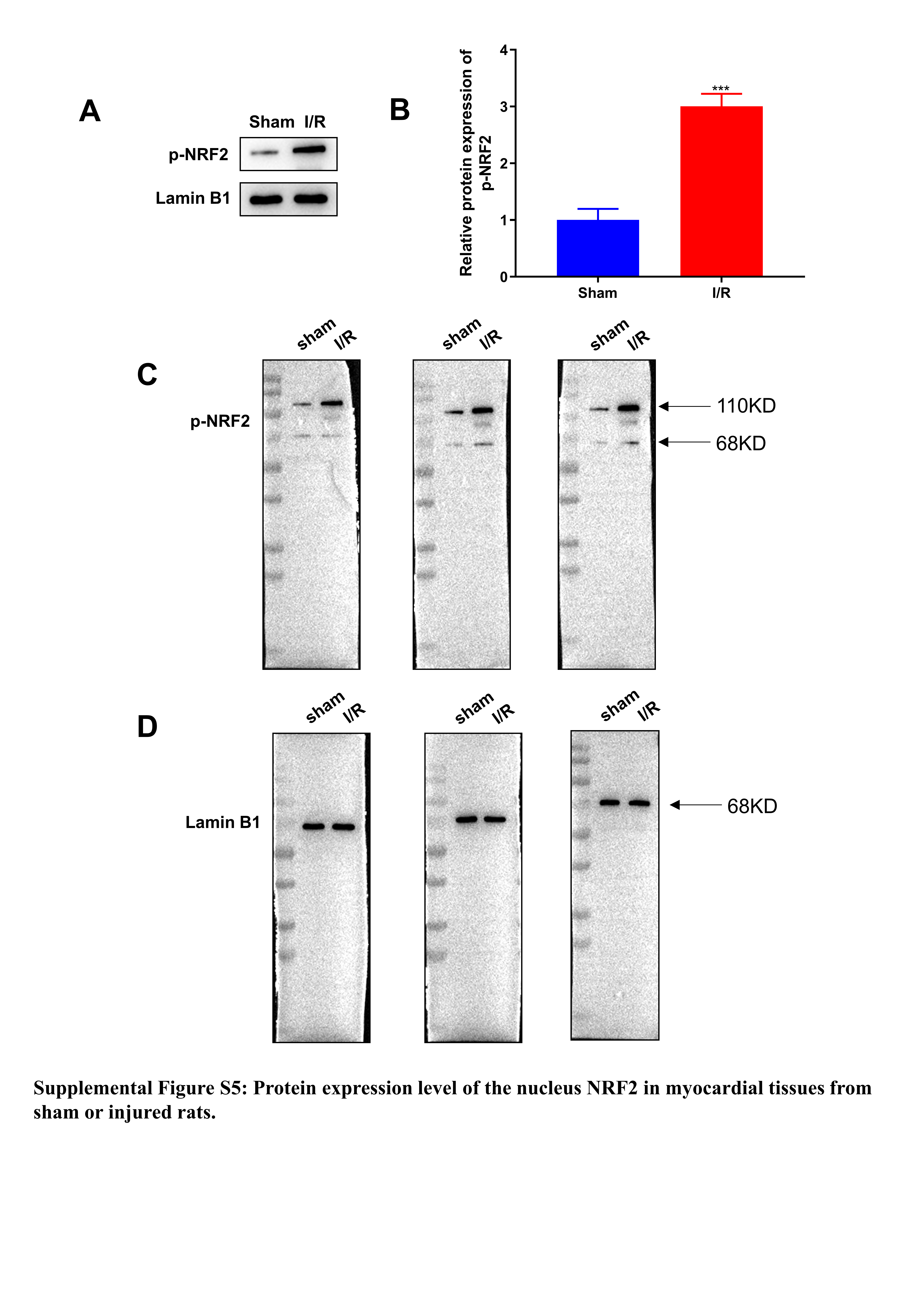

Supplement: Supplementary file 7 [file Image5.TIF]
