# Supplementary material for: LncAABR07025387.1 Enhances Myocardial Ischemia/Reperfusion Injury Via miR-205/ACSL4-Mediated Ferroptosis
Source: Front Cell Dev Biol. 2022 Feb 2;10:672391. doi: 10.3389/fcell.2022.672391 (PMC8847229; doi:10.3389/fcell.2022.672391)
Supplement: Supplementary file 3 [file Image4.PDF]

Figure1-K

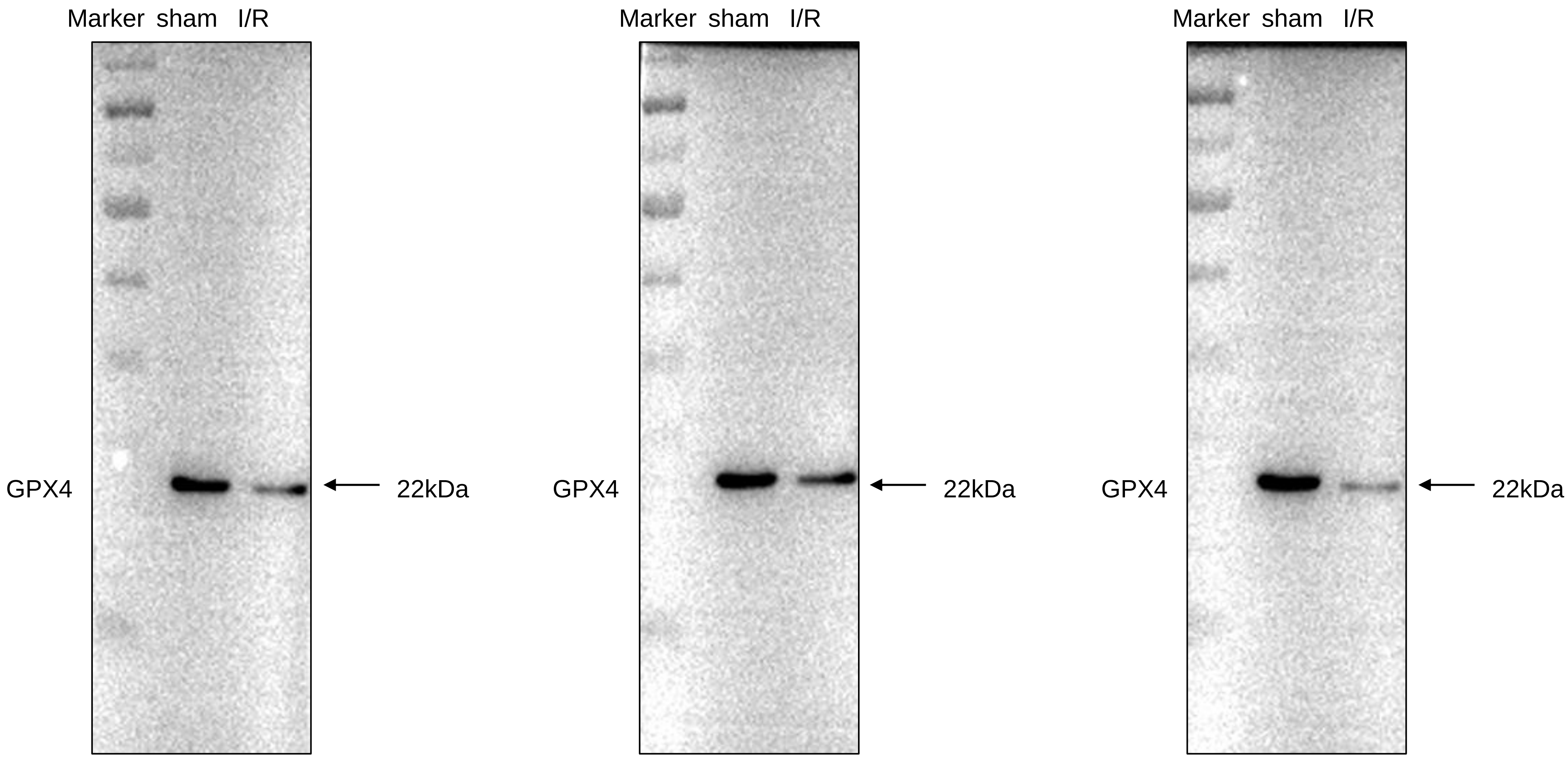
Marker sham I/R
GPX4
22kDa
Marker sham I/R
GPX4
22kDa
Marker sham I/R
GPX4
22kDa

Figure1-K

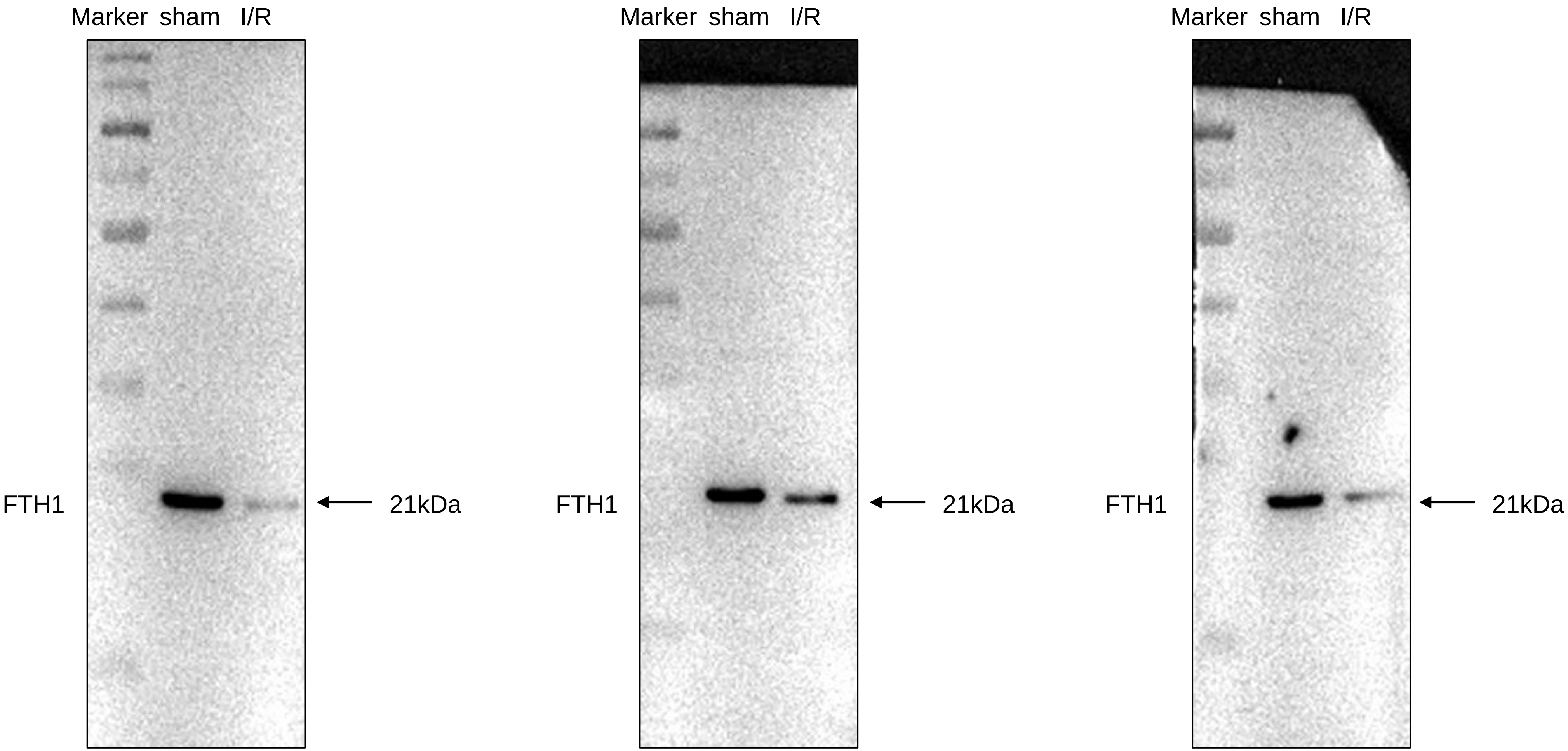

Marker sham I/R
FTH1
21kDa
Marker sham I/R
FTH1
21kDa
Marker sham I/R
FTH1
21kDa

Figure1-K

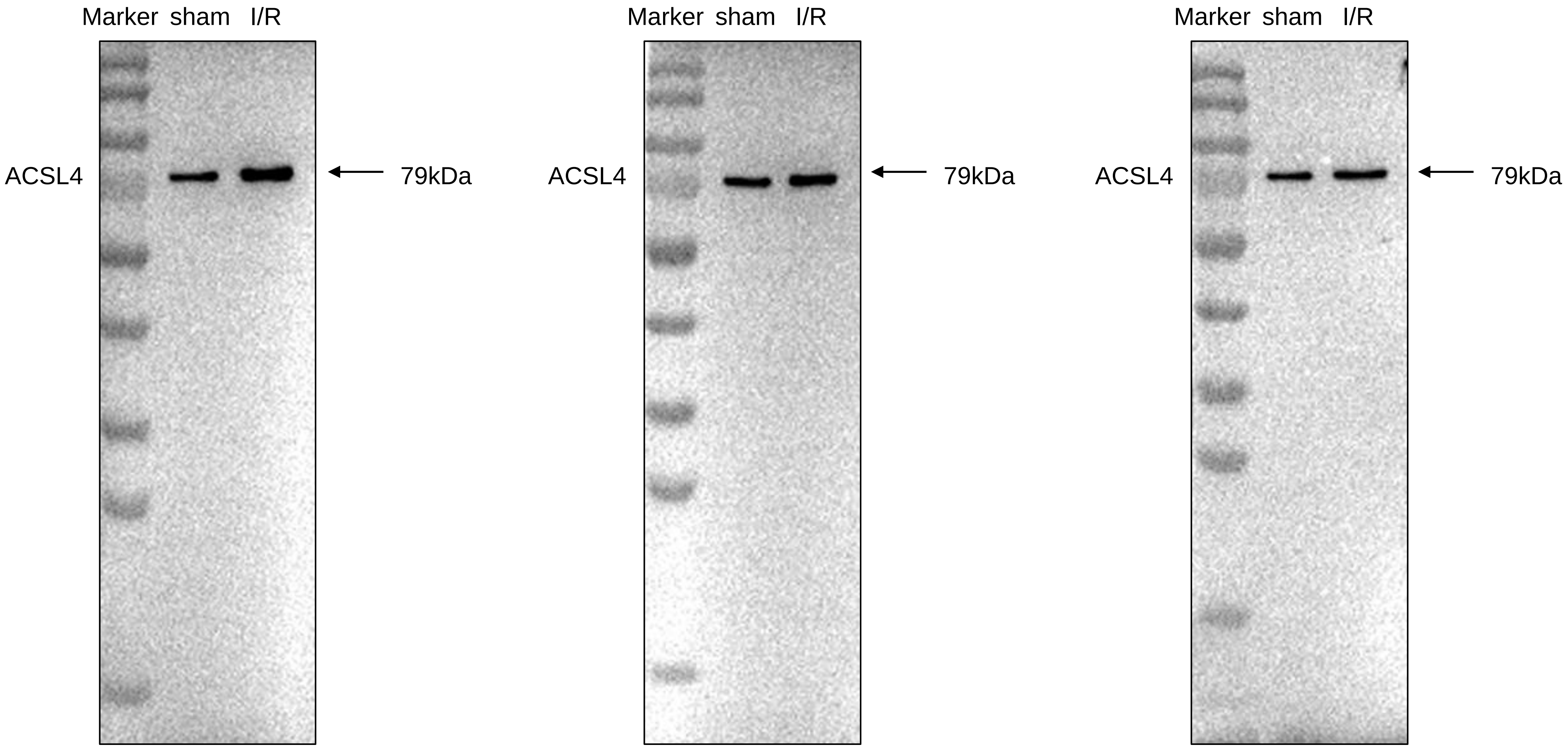
Marker sham I/R
ACSL4
79kDa
Marker sham I/R
ACSL4
79kDa
Marker sham I/R
ACSL4
79kDa

Figure1-K

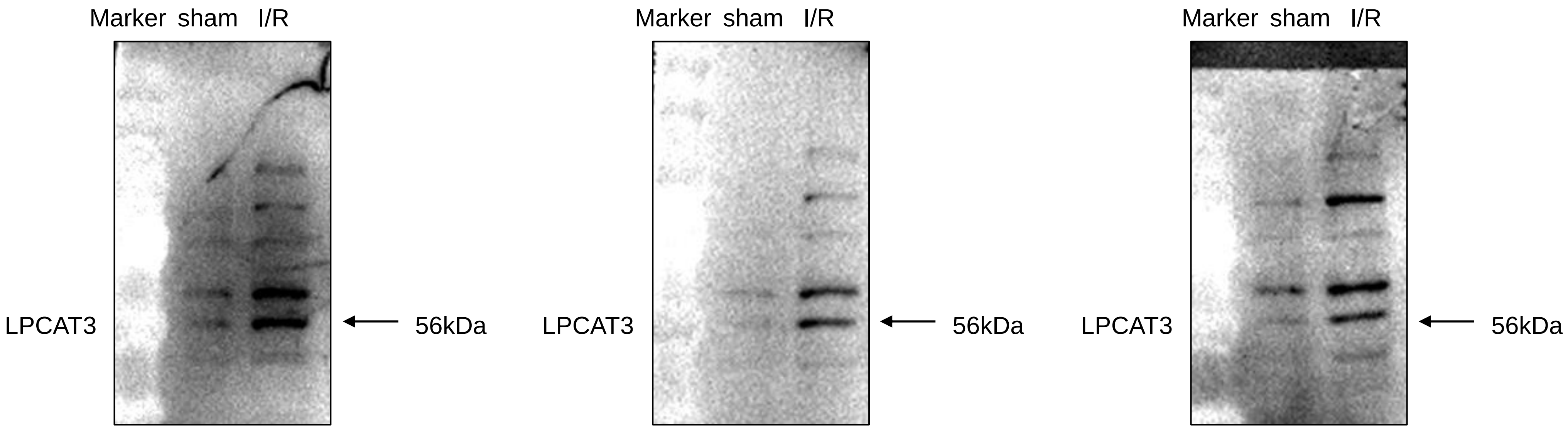

Marker sham I/R
LPCAT3
56kDa
Marker sham I/R
LPCAT3
56kDa
Marker sham I/R
LPCAT3
56kDa

Figure1-K

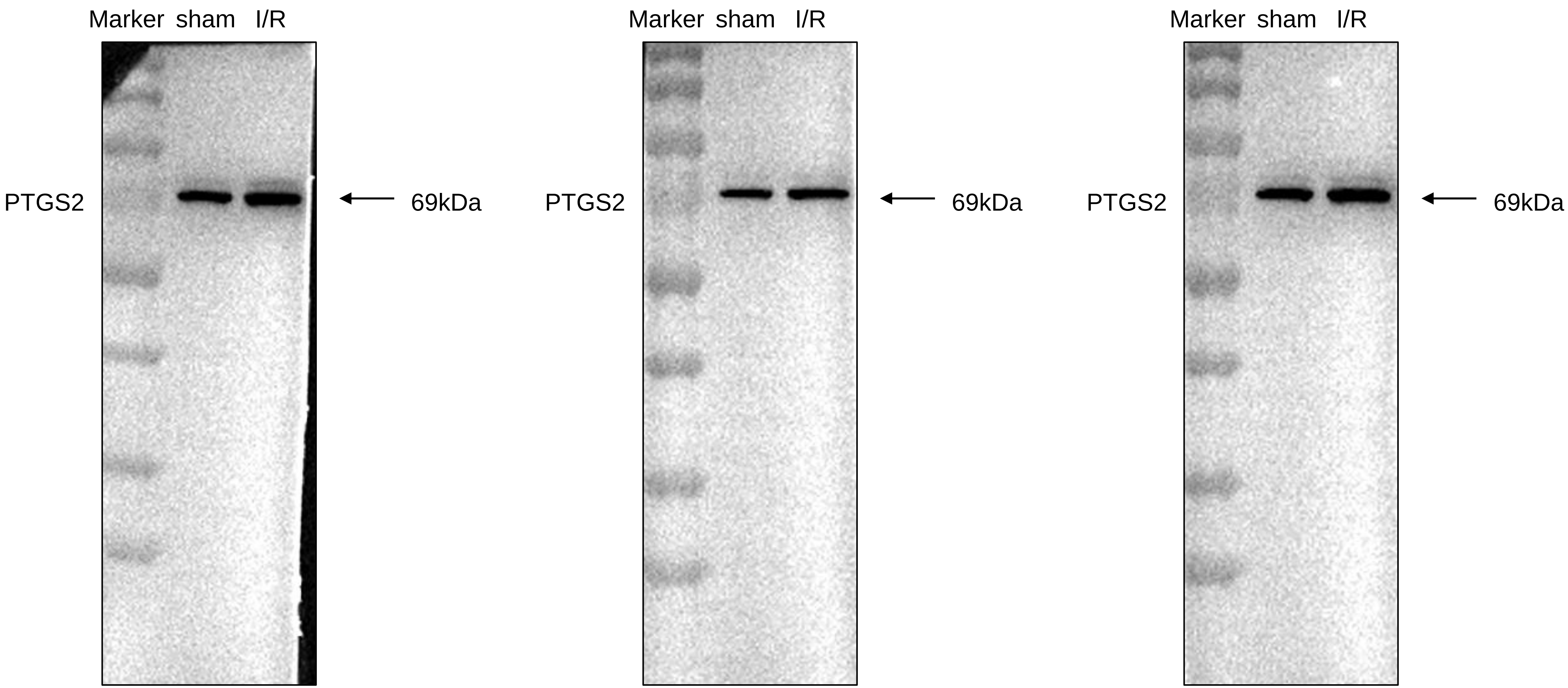
Marker sham I/R
PTGS2
69kDa
Marker sham I/R
PTGS2
69kDa
Marker sham I/R
PTGS2
69kDa

Figure1-K

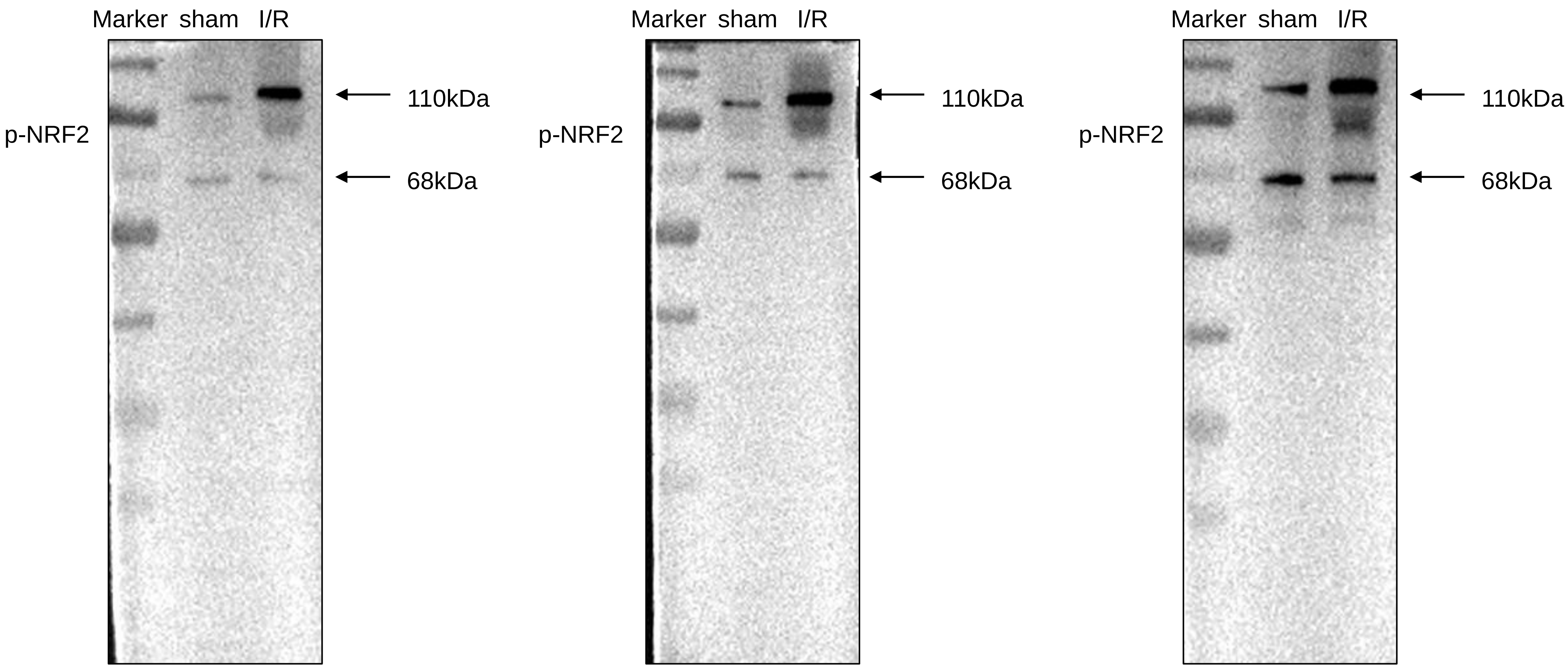
Marker sham I/R
p-NRF2
110kDa
68kDa
Marker sham I/R
p-NRF2
110kDa
68kDa
Marker sham I/R
p-NRF2
110kDa
68kDa

Figure1-K

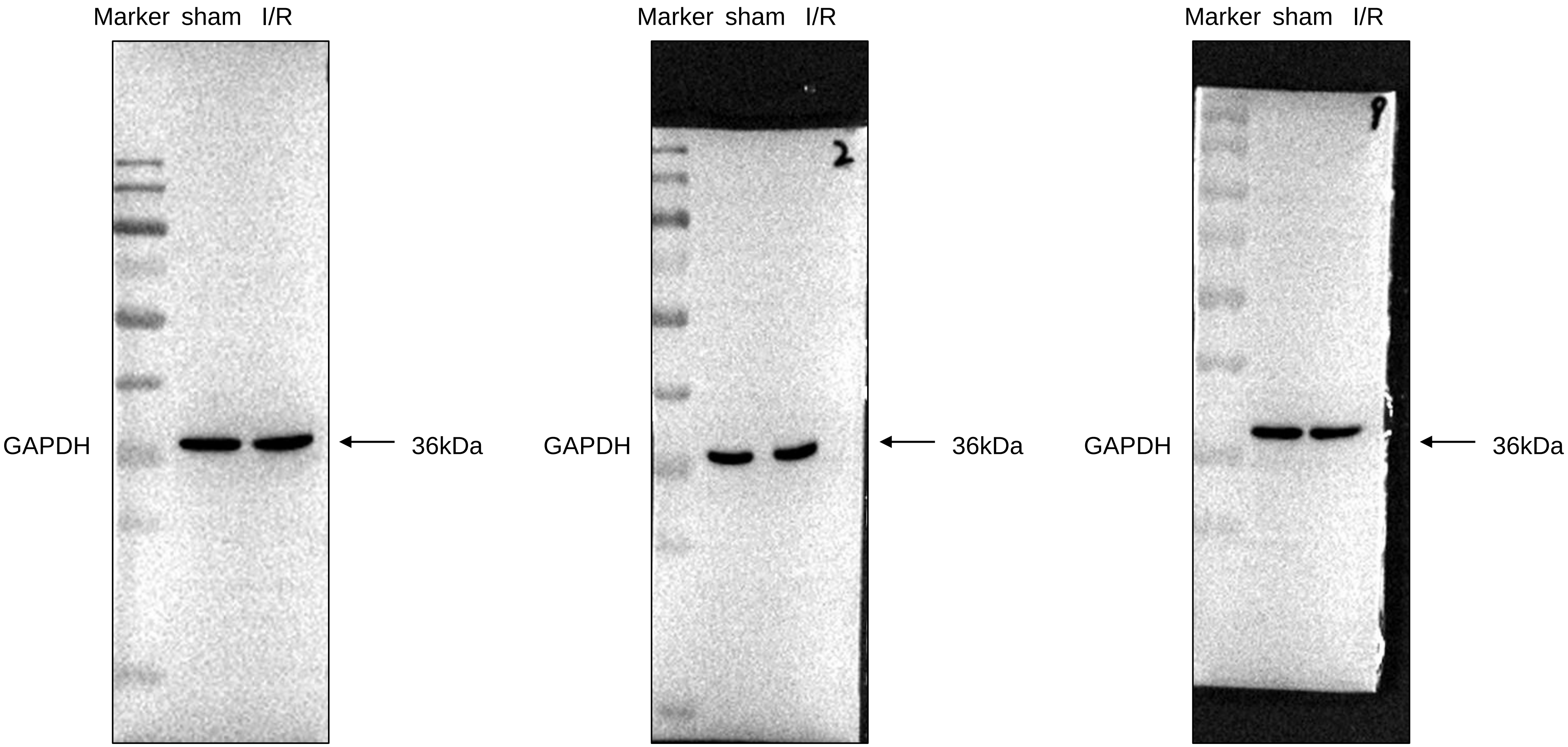
Marker sham I/R
GAPDH
36kDa
Marker sham I/R
GAPDH
36kDa
Marker sham I/R
GAPDH
36kDa

Figure3-E

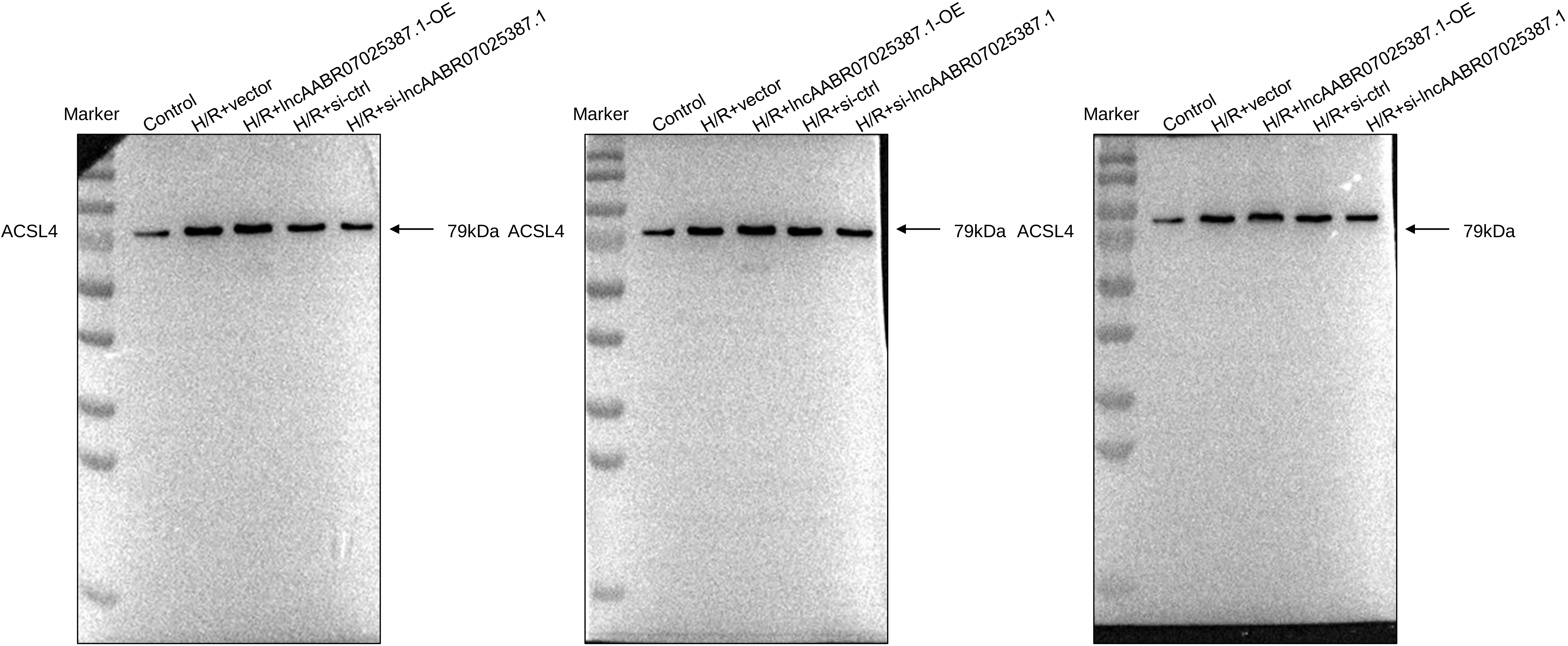
Marker
Control
H/R+vector
H/R+lncAABR07025387.1-OE
H/R+si-ctrl
H/R+si-lncAABR07025387.1
ACSL4
79kDa
Marker
Control
H/R+vector
H/R+lncAABR07025387.1-OE
H/R+si-ctrl
H/R+si-lncAABR07025387.1
ACSL4
79kDa
Marker
Control
H/R+vector
H/R+lncAABR07025387.1-OE
H/R+si-ctrl
H/R+si-lncAABR07025387.1
ACSL4
79kDa

Figure3-E

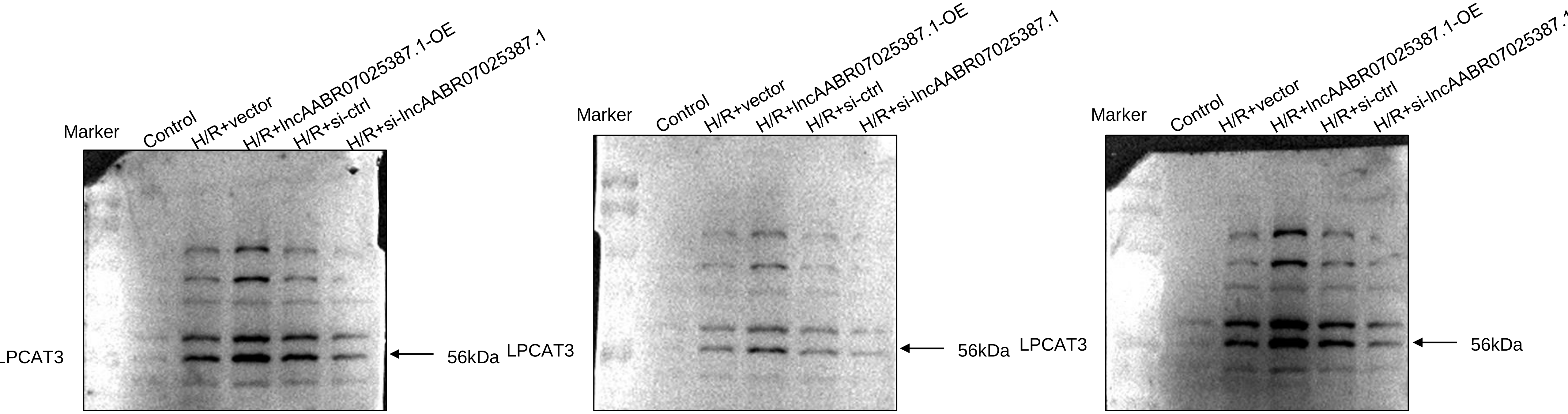

Marker
Control
H/R+vector
H/R+lncAABR07025387.1-OE
H/R+si-ctrl
H/R+si-lncAABR07025387.1
LPCAT3
56kDa
Marker
Control
H/R+vector
H/R+lncAABR07025387.1-OE
H/R+si-ctrl
H/R+si-lncAABR07025387.1
LPCAT3
56kDa
Marker
Control
H/R+vector
H/R+lncAABR07025387.1-OE
H/R+si-ctrl
H/R+si-lncAABR07025387.
LPCAT3
56kDa

Figure3-E

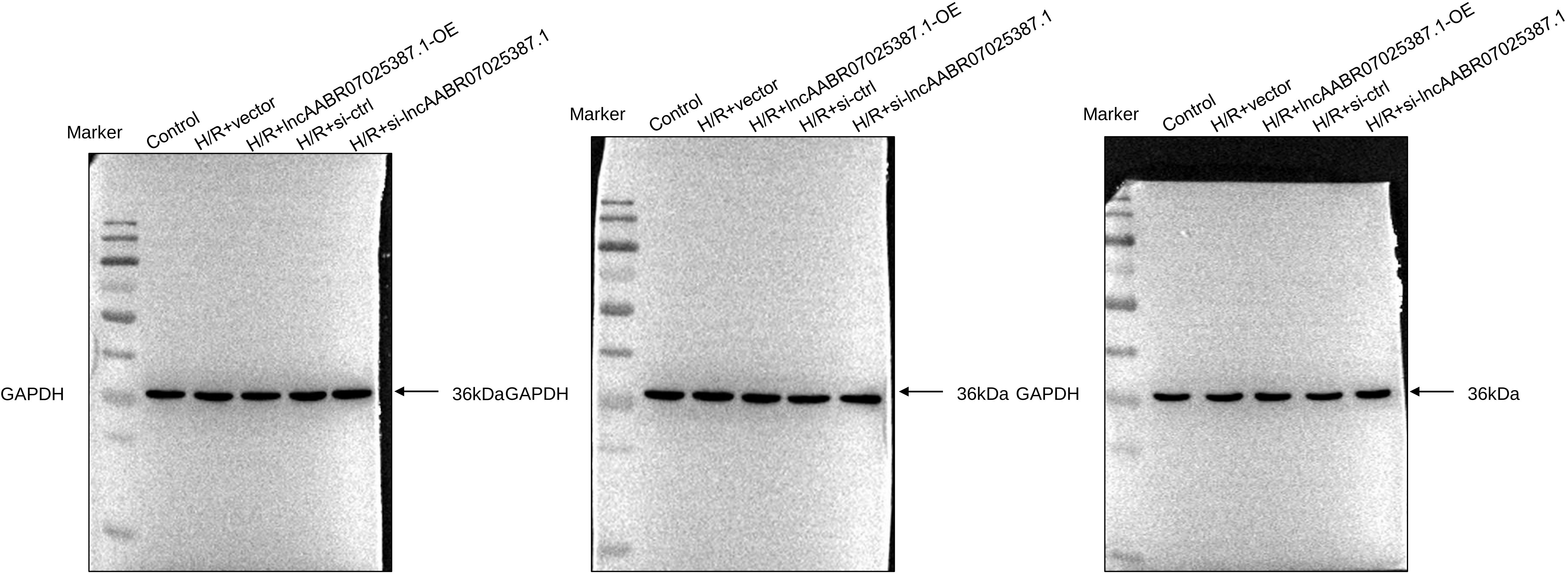
Marker
Control
H/R+vector
H/R+lncAABR07025387.1-OE
H/R+si-ctrl
H/R+si-lncAABR07025387.1
GAPDH
36kDa
GAPDH
36kDa
GAPDH
36kDa

Figure5-E

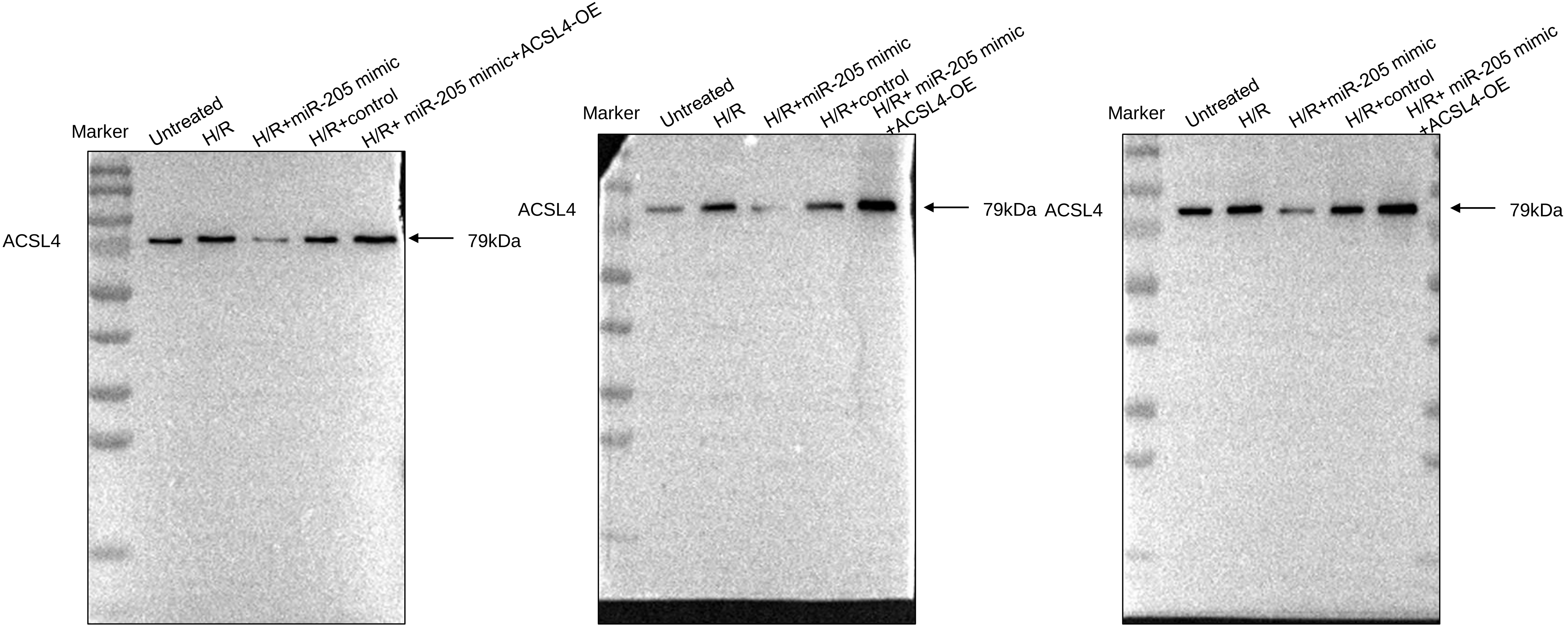
Marker
Untreated
H/R
H/R+miR-205 mimic
H/R+control
H/R+ miR-205 mimic+ACSL4-OE
ACSL4
79kDa
Marker
Untreated
H/R
H/R+miR-205 mimic
H/R+control
H/R+ miR-205 mimic
+ACSL4-OE
ACSL4
79kDa
Marker
Untreated
H/R
H/R+miR-205 mimic
H/R+control
H/R+ miR-205 mimic
+ACSL4-OE
ACSL4
79kDa

Figure5-E

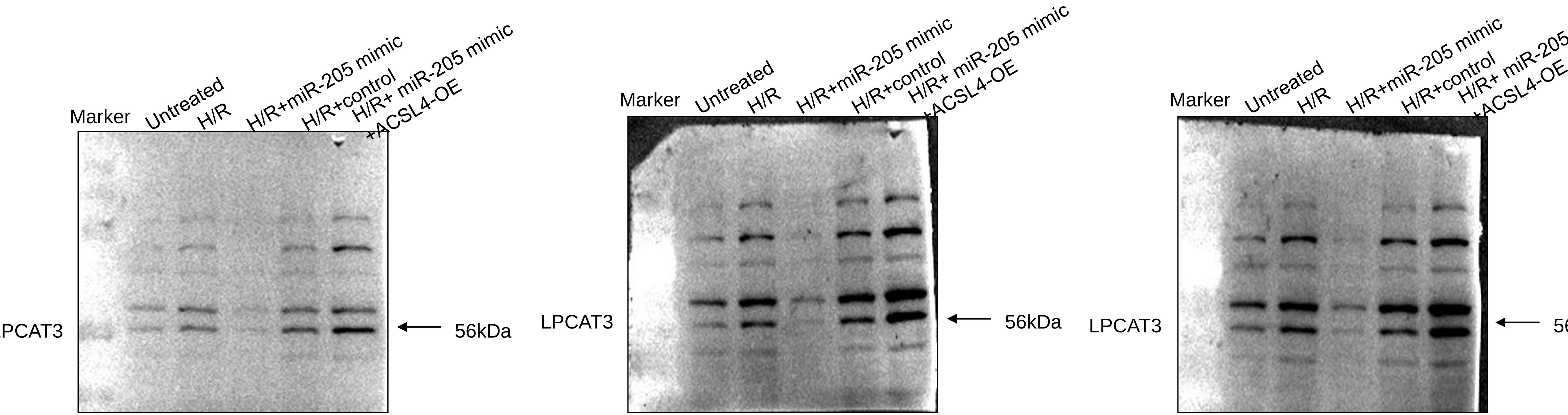
Marker
Untreated
H/R
H/R+miR-205 mimic
H/R+control
H/R+ miR-205 mimic
+ACSL4-OE
PCAT3
56kDa
Marker
Untreated
H/R
H/R+miR-205 mimic
H/R+control
H/R+ miR-205 mimic
+ACSL4-OE
LPCAT3
56kDa
Marker
Untreated
H/R
H/R+miR-205 mimic
H/R+control
H/R+ miR-205
+ACSL4-OE
LPCAT3

Figure5-E

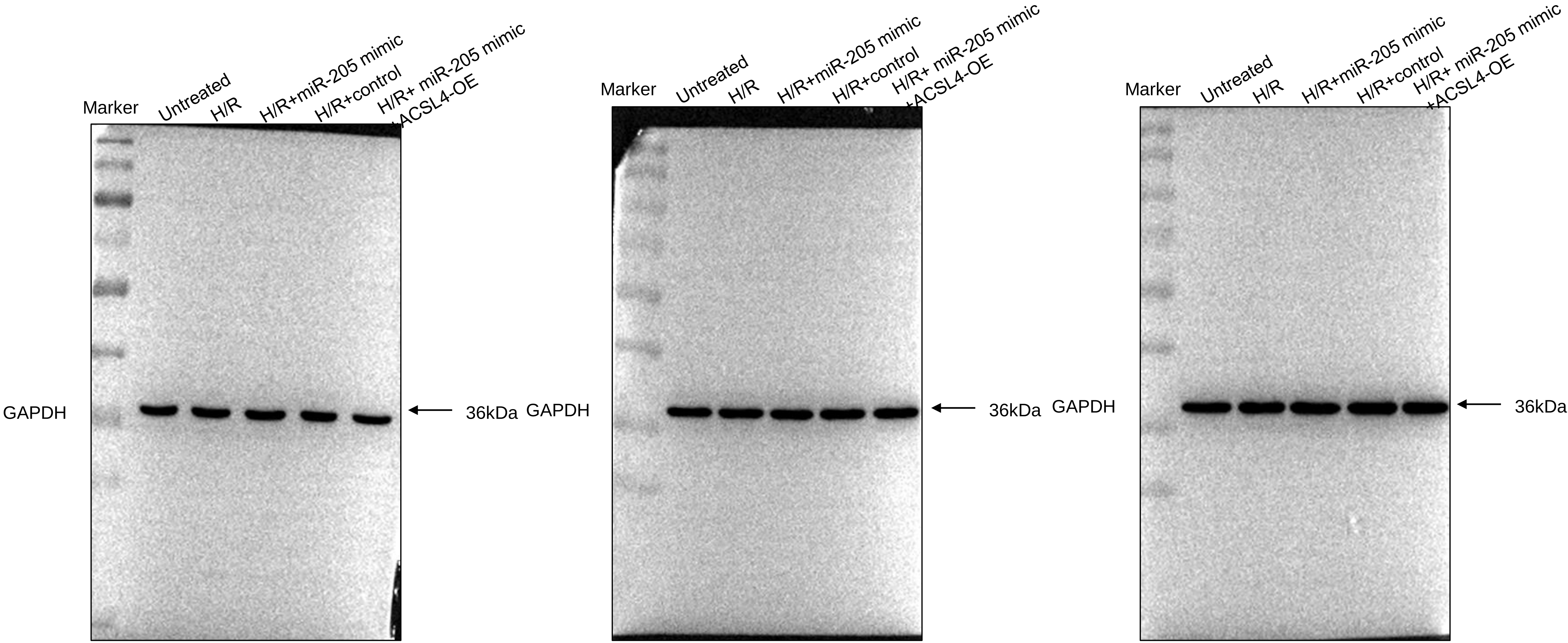
Marker
Untreated
H/R
H/R+miR-205 mimic
H/R+control
H/R+ miR-205 mimic
+ACSL4-OE
GAPDH
36kDa
Marker
Untreated
H/R
H/R+miR-205 mimic
H/R+control
H/R+ miR-205 mimic
+ACSL4-OE
GAPDH
36kDa
Marker
Untreated
H/R
H/R+miR-205 mimic
H/R+control
H/R+ miR-205 mimic
+ACSL4-OE
GAPDH
36kDa

Figure7-C

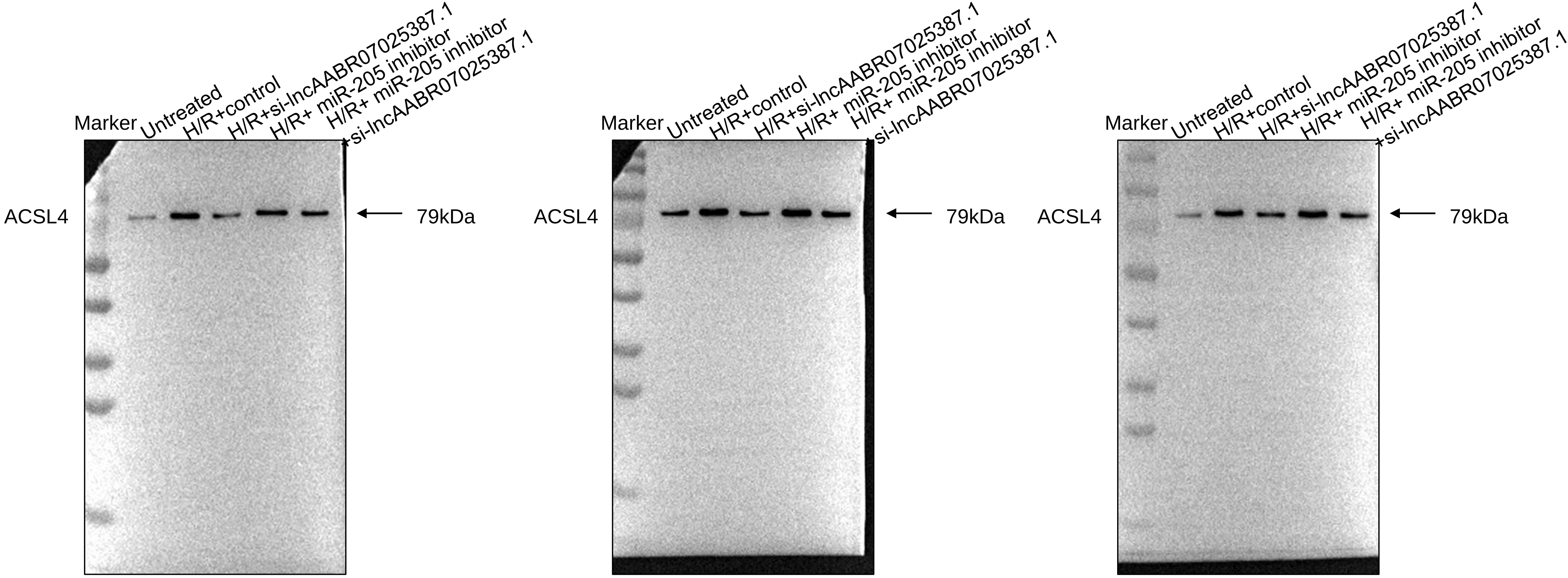
Marker
Untreated
H/R+control
H/R+si-lncAABR07025387.1
H/R+ miR-205 inhibitor
H/R+ miR-205 inhibitor
+si-lncAABR07025387.1
ACSL4
79kDa
Marker
Untreated
H/R+control
H/R+si-lncAABR07025387.1
H/R+ miR-205 inhibitor
H/R+ miR-205 inhibitor
+si-lncAABR07025387.1
ACSL4
79kDa
Marker
Untreated
H/R+control
H/R+si-lncAABR07025387.1
H/R+ miR-205 inhibitor
H/R+ miR-205 inhibitor
+si-lncAABR07025387.1
ACSL4
79kDa

Figure7-C

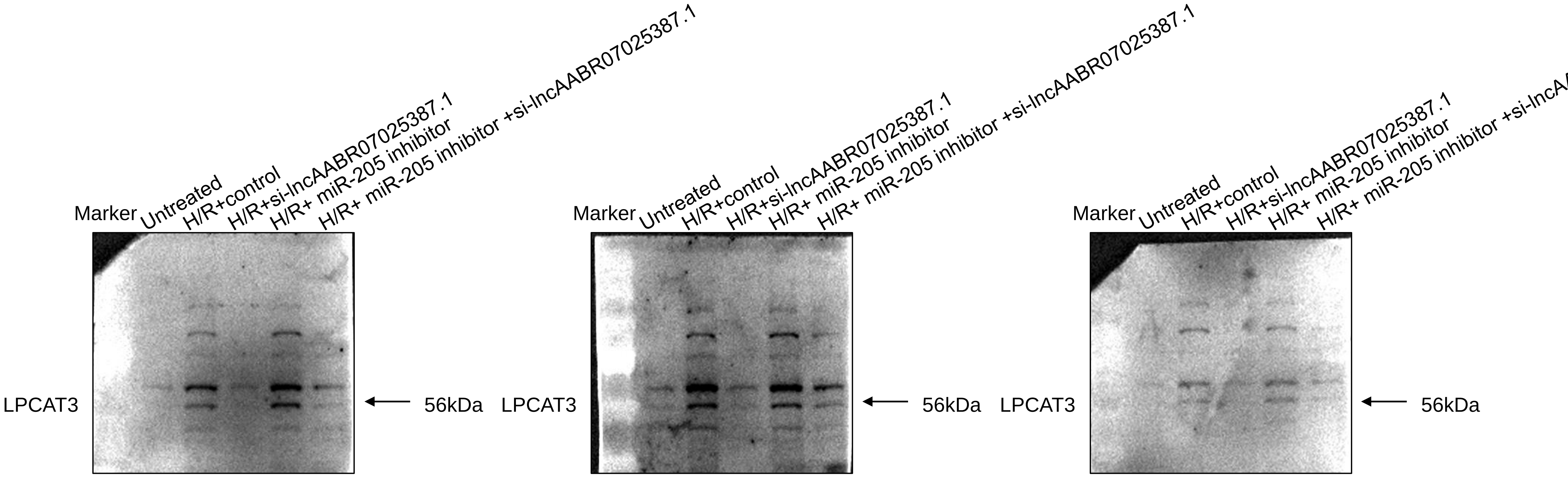
Marker
Untreated
H/R+control
H/R+si-lncAABR07025387.1
H/R+ miR-205 inhibitor
H/R+ miR-205 inhibitor +si-lncAABR07025387.1
LPCAT3
56kDa
Marker
Untreated
H/R+control
H/R+si-lncAABR07025387.1
H/R+ miR-205 inhibitor
H/R+ miR-205 inhibitor +si-lncAABR07025387.1
LPCAT3
56kDa
Marker
Untreated
H/R+control
H/R+si-lncAABR07025387.1
H/R+ miR-205 inhibitor
H/R+ miR-205 inhibitor +si-lncA
LPCAT3
56kDa

Figure7-C

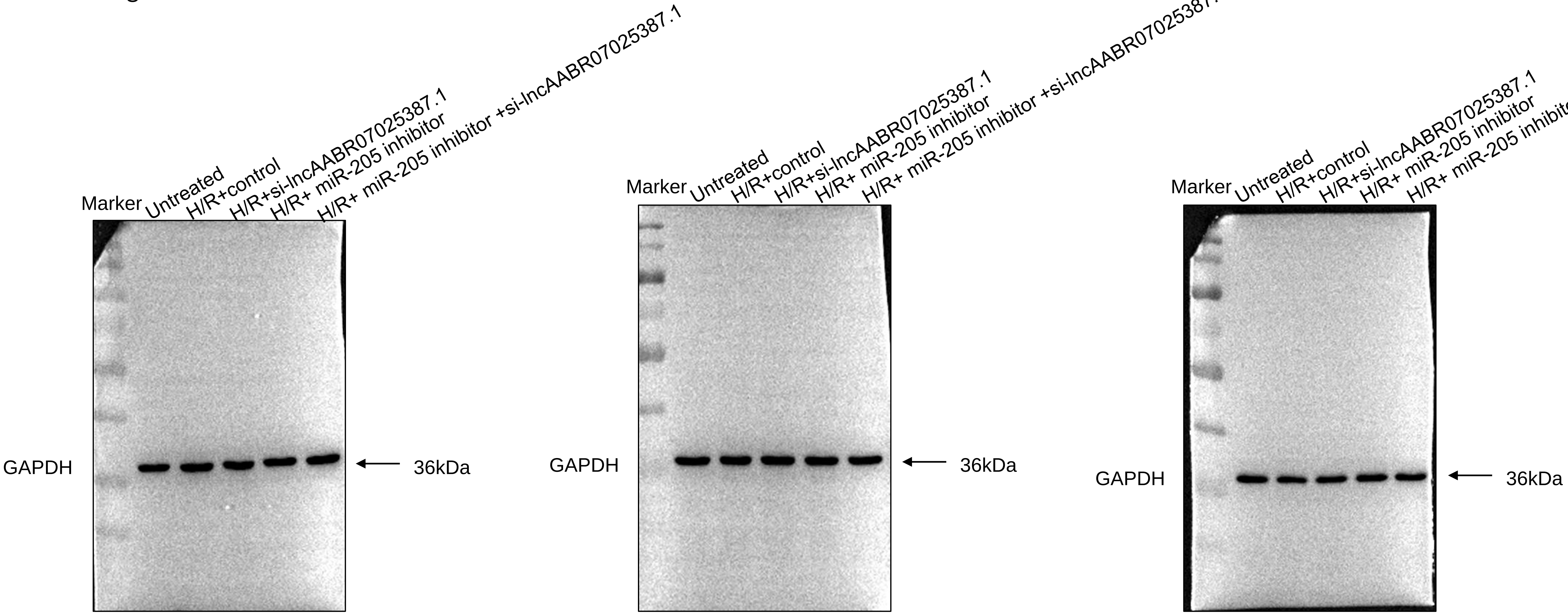
Marker
Untreated
H/R+control
H/R+si-lncAABR07025387.1
H/R+ miR-205 inhibitor
H/R+ miR-205 inhibitor +si-lncAABR07025387.1
GAPDH
36kDa
Marker
Untreated
H/R+control
H/R+si-lncAABR07025387.1
H/R+ miR-205 inhibitor
H/R+ miR-205 inhibitor +si-lncAABR07025387.1
GAPDH
36kDa
Marker
Untreated
H/R+control
H/R+si-lncAABR07025387.1
H/R+ miR-205 inhibitor
GAPDH
36kDa

Figure8-G

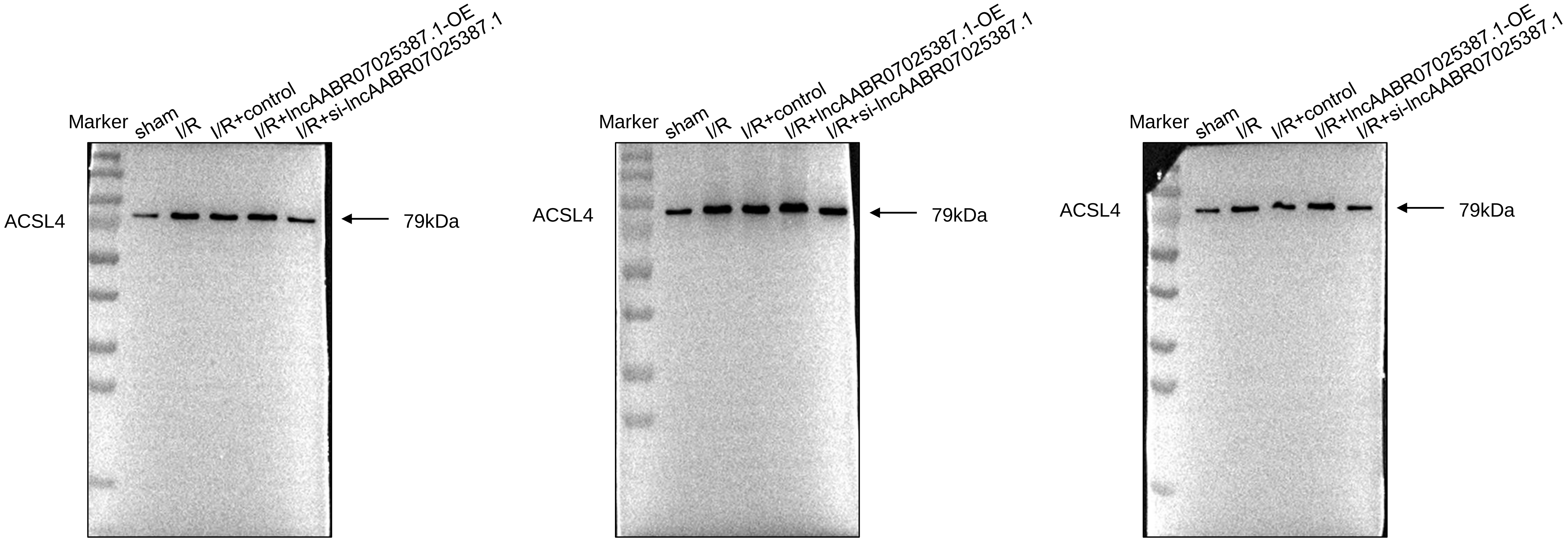

Marker
sham
I/R
I/R+control
I/R+lncAABR07025387.1-OE
I/R+si-lncAABR07025387.1
ACSL4
79kDa
Marker
sham
I/R
I/R+control
I/R+lncAABR07025387.1-OE
I/R+si-lncAABR07025387.1
ACSL4
79kDa
Marker
sham
I/R
I/R+control
I/R+lncAABR07025387.1-OE
I/R+si-lncAABR07025387.1
ACSL4
79kDa

Figure8-G

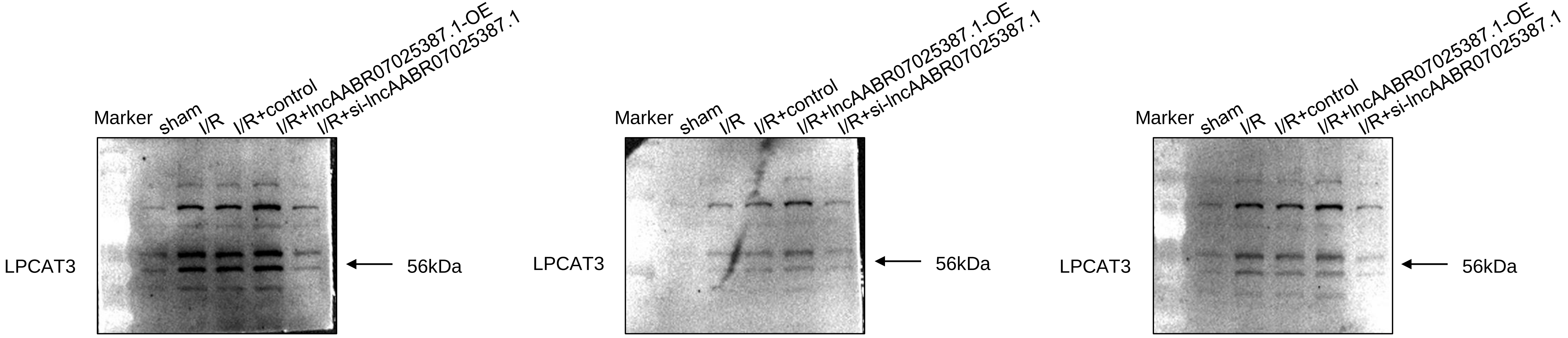
Marker
sham
I/R
I/R+control
I/R+lncAABR07025387.1-OE
I/R+si-lncAABR07025387.1
LPCAT3
56kDa
Marker
sham
I/R
I/R+control
I/R+lncAABR07025387.1-OE
I/R+si-lncAABR07025387.1
LPCAT3
56kDa
Marker
sham
I/R
I/R+control
I/R+lncAABR07025387.1-OE
I/R+si-lncAABR07025387.1
LPCAT3
56kDa

Figure8-G

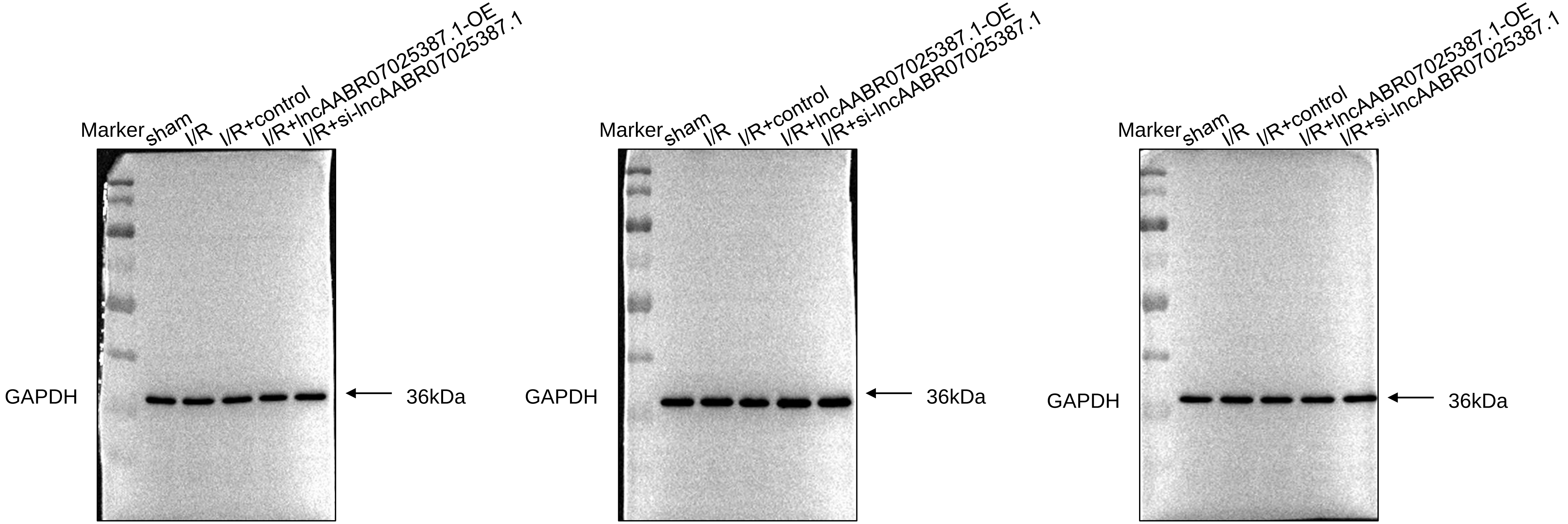
Marker
sham
I/R
I/R+control
I/R+lncAABR07025387.1-OE
I/R+si-lncAABR07025387.1
GAPDH
36kDa
Marker
sham
I/R
I/R+control
I/R+lncAABR07025387.1-OE
I/R+si-lncAABR07025387.1
GAPDH
36kDa
Marker
sham
I/R
I/R+control
I/R+lncAABR07025387.1-OE
I/R+si-lncAABR07025387.1
GAPDH
36kDa
