# Supplementary material for: LncAABR07025387.1 Enhances Myocardial Ischemia/Reperfusion Injury Via miR-205/ACSL4-Mediated Ferroptosis
Source: Front Cell Dev Biol. 2022 Feb 2;10:672391. doi: 10.3389/fcell.2022.672391 (PMC8847229; doi:10.3389/fcell.2022.672391)
Supplement: Supplementary file 4 [file Image2.PDF]

Supplementary Figure2: The schematic diagram of experimental animals grouped and surgically molded for survival

Animal Experiments Part I
（Figure1）

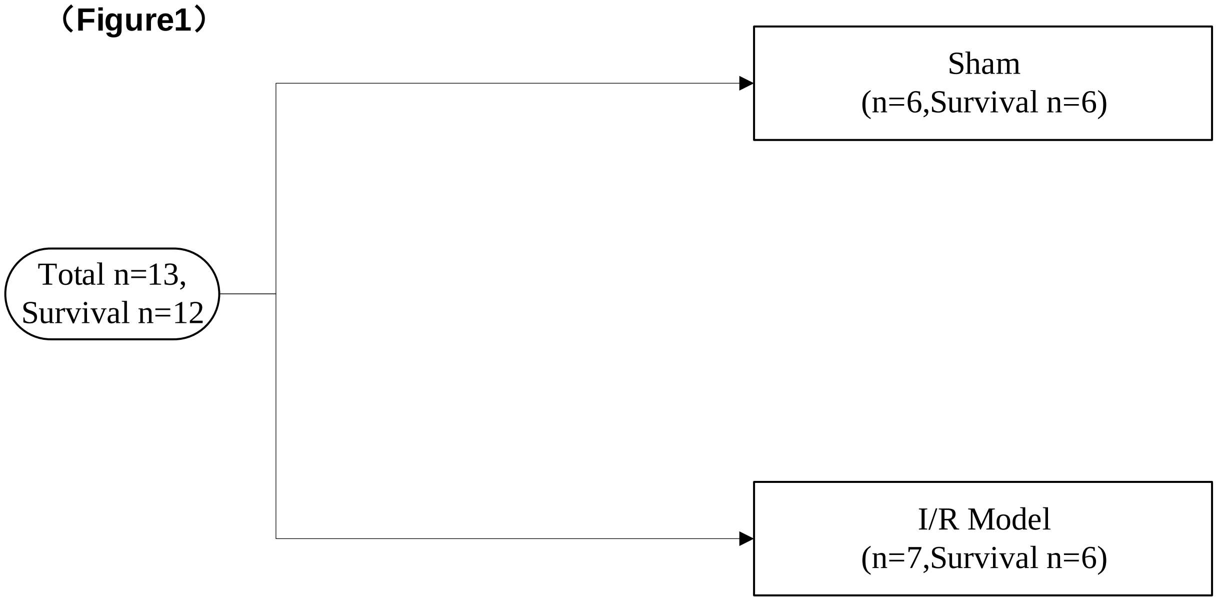

Animal Experiments Part 2
（Figure8）

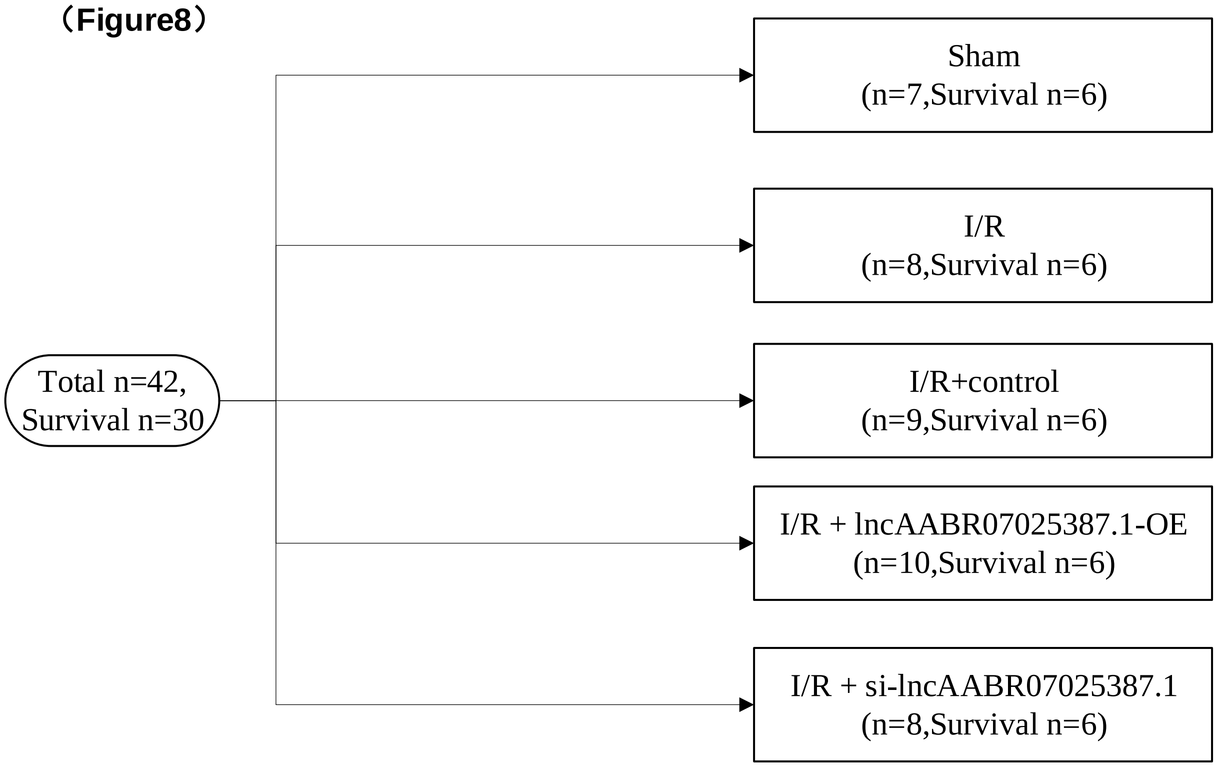

In order to end up with 6 rats in each group for the final study, we estimated the experimental animals based on a survival rate of approximately 20-30% for the rat molds and contacted the animal experimentation center managers to continue managing the rats that did not enter the experiment. If there are not enough surviving animals after surgery, they will continue to be replenished until there are 6 rats in each group.
Experiment part 1 prepared 18 rats (6×2÷0.7=17.1), and 13 rats actually entered the experiment. Experiment part 2 prepared 43 rats (6×5÷0.7=42.85), and 42 rats actually entered the experiment.
