# Supplementary material for: LncAABR07025387.1 Enhances Myocardial Ischemia/Reperfusion Injury Via miR-205/ACSL4-Mediated Ferroptosis
Source: Front Cell Dev Biol. 2022 Feb 2;10:672391. doi: 10.3389/fcell.2022.672391 (PMC8847229; doi:10.3389/fcell.2022.672391)
Supplement: Supplementary file 5 [file Image3.PDF]

Supplementary Figure S3: The flow chart of constructing myocardial ischemia-reperfusion injury model in rats

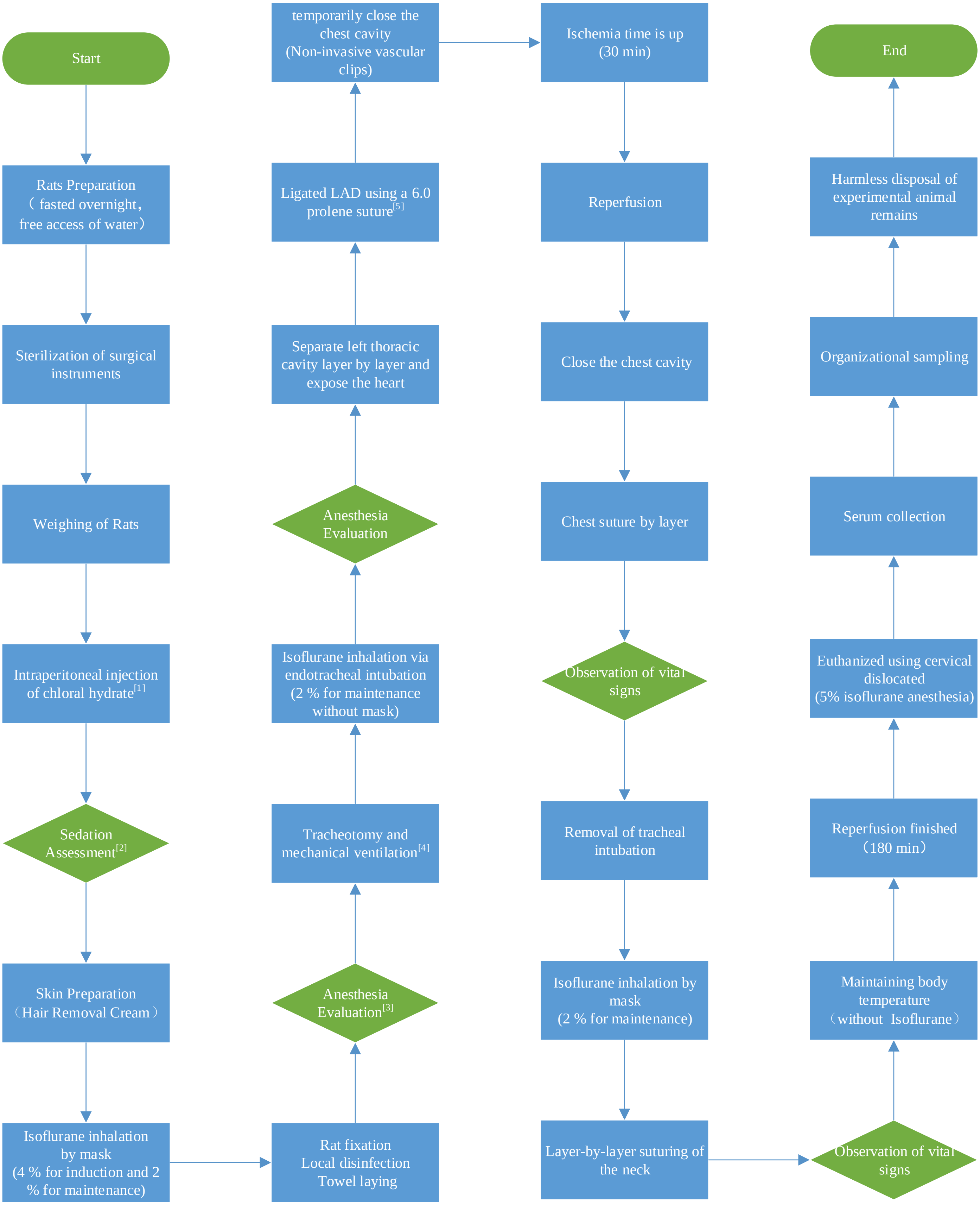

[1] 0.5ml/100g (5% chloral hydrate).25% additional first dose for unsatisfactory sedation.(PS:The maximum dose is 300 mg/kg . The LD50 is 480mg/kg.)
[2] Reduced rollover reflex in rats.
[3] No pain reflex
[4] Cut with a 10 ml syringe needle. Rat Mode, Tidal volume:2ml/100g, Breathing rate:80/min, Exhalation and inhalation ratio：3:4~1:2.
[5]A small semi-cylindrical plastic hose was inserted to the LAD to facilitate the opening of the knot during reperfusion and to reduce the mechanical damage to the heart tissue and blood vessels.
