# Supplementary material for: LncAABR07025387.1 Enhances Myocardial Ischemia/Reperfusion Injury Via miR-205/ACSL4-Mediated Ferroptosis
Source: Front Cell Dev Biol. 2022 Feb 2;10:672391. doi: 10.3389/fcell.2022.672391 (PMC8847229; doi:10.3389/fcell.2022.672391)
Supplement: Supplementary file 6 [file Table2.DOCX]

**Table S2. Primer sequences of genes in RT-qPCR assay**

| **Gene** | **Forward Primer** | **Reversed Primer** |
| --- | --- | --- |
| GPX4 (rat) | 5'-GCAGGCACCACTTCTAAACC-3' | 5'-GGGAGACAACTCGTTCAGAC-3' |
| FTH1 (rat) | 5'-GGCGACGGAAGTGGTTGTTA-3' | 5'-ACAGGGTTACTGGTCAGCTCT-3' |
| ACSL4 (rat) | 5'- ACCTTCGGTGAGTGATCCTA-3' | 5'-CGGGATACAGGGAAAACCCA-3' |
| LPCAT3 (rat) | 5'-GGGAAATAGGCAGAATGGGGA-3' | 5'-CGATAGCTGGCTGACGTACT-3' |
| PTGS2 (rat) | 5'-AGAACCGCATTGCCTCTGAA-3' | 5'-AGGAACAATGCTTACCCGGC-3' |
| NRF2 (rat) | 5'-CCCCTCAGCTATGCCAAGAAT-3' | 5'-GCTAGGACCCCACAATCGTT-3' |
| GAPDH (rat) | 5'-TGAGGACCAGGTTGTCTCCT-3' | 5'-GAGGGCACCAAACCTTCAGTT-3' |
| lncAABR07025387.1 (rat) | 5'-CCAGCAACCCTAACGGAGAT-3' | 5'-CTGAGCATAGTGCTGCCCAA-3' |
| lncAABR07017145.1 (rat) | 5'-TCTGAAAGCCCACTGAAGCAT-3' | 5'-GCCCAACCTCAGATCCTGAAT-3' |
| lncBves (rat) | 5'-TGCTTCAGTCCTGCCAGATG-3' | 5'-TCTACTCAGAAGGCTCGGCT-3' |
| lncLOC100364190 (rat) | 5'-ACTGACCCTTGGCACCTAGA-3' | 5'-GCGATGACCCCTCCAATGAT-3' |
| lncRn60_1_2682.1 (rat) | 5'-GGCGAGGCTAATGATGTGGTA-3' | 5'-TTAGGGGCATCTGTTTGCCA-3' |
| rno-miR-330-3p | 5'-GGCAAAGCACAGGGCCTG-3' | 5'-AGTGCAGGGTCCGAGGTATT-3' |
| rno-miR-296-3p | 5'-CGGAGGGTTGGGTGGAGG-3' | 5'-AGTGCAGGGTCCGAGGTATT-3' |
| rno-miR-140-3p | 5'-GCGCGTACCACAGGGTAGAA-3' | 5'-AGTGCAGGGTCCGAGGTATT-3' |
| rno-miR-10b-3p | 5'-GCGCGACAGATTCGATTCTA-3' | 5'-AGTGCAGGGTCCGAGGTATT-3' |
| rno-miR-205 | 5'-CGTCCTTCATTCCACCGGA-3' | 5'-AGTGCAGGGTCCGAGGTATT-3' |
| rno-miR-3588 | 5'-GCGCGTCACAAGTTAGGGTCT-3' | 5'-AGTGCAGGGTCCGAGGTATT-3' |
| U6 (rat) | 5’-GCTCGCTTCGGCAGCACAT-3’ | 5’-ATGGAACGCTTCACGAAT-3’ |
